# The cyclooxygenase-2 upregulation mediates production of PGE2 autacoid to positively regulate interleukin-6 secretion in chronic rhinosinusitis with nasal polyps and polyp-derived fibroblasts

**DOI:** 10.1038/s41598-024-58143-2

**Published:** 2024-03-30

**Authors:** Jiunn-Min Shieh, Yih-Jeng Tsai, Ming-Chieh Ma, Chih-Li Chen, Wen-Bin Wu

**Affiliations:** 1 Department of Internal Medicine, Chi-Mei Medical Center, Tainan, Taiwan; 2 School of Medicine, Fu Jen Catholic University, No.510, Zhongzheng Rd., Xinzhuang Dist., New Taipei City, 242062 Taiwan; 3 Department of Otolaryngology Head and Neck Surgery, Shin Kong Wu Ho Su Memorial Hospital, Taipei, Taiwan; 4 Graduate Institute of Biomedical and Pharmaceutical Science, Fu Jen Catholic University, New Taipei City, Taiwan

**Keywords:** Cell signalling, Mechanisms of disease, Biomarkers, Molecular medicine

## Abstract

Chronic rhinosinusitis (CRS) can be traditionally classified as CRSwNP [with nasal polyps (NPs)] and CRSsNP (without NPs) based on the clinical phenotypes but recently suggested to be classified by the endotypes. We have identified overexpression of the *cyclooxygenase-2* (*COX-2*) gene in NP tissues of Taiwanese CRSwNP patients. Therefore, in this study, we sought to investigate its protein expression/location/distribution in NP specimens and explore its roles in nasal polyposis. The COX-2 protein and mRNA expression was found higher in NPs than that in the control and CRSsNP patients’ nasal tissues, mainly located at the epithelium and subepithelial stroma. Consistently, the CRS-related peptidoglycan (PGN) and bradykinin provoked COX-2 mRNA and protein upregulation in the human NP-derived fibroblasts and caused PGE_2_, thromboxane A_2_ (TXA_2_), and interleukin (IL-6) secretion in culture medium. Further analysis revealed that the PI3K/Akt activation and COX-2 induction were necessarily required for PGN-induced IL-6 production/secretion and the induced PGE_2_, but not TXA_2_, was speculated to affect IL-6 protein trafficking and production. Finally, the IL-6 increase observed in vitro could also be detected in NP tissues. Collectively, we demonstrated here that COX-2 protein and IL-6 are overexpressed in human NP tissues. In response to PGN challenge, the PI3K/Akt activation and COX-2-mediated PGE_2_ autacoid correlates with extracellular IL-6 protein trafficking/production in NP-derived fibroblasts, which can additionally contribute to the production of Th17-related cytokines such as IL-17 and TNF-α. This study also suggests COX-2 as a special biomarker for CRSwNP endotyping and may highlight the importance of COX-2 inhibitors in treating CRSwNP.

## Introduction

Rhinosinusitis (RS) is defined as the inflammation of the nasal cavity and paranasal sinuses and is associated with symptoms such as nasal congestion, nasal secretion, etc. If inflammation and symptoms persist for over 12 weeks, the presence of chronic rhinosinusitis (CRS) is confirmed^1^. CRS causes a total of 11.5 million workdays missed and the range of overall CRS-related healthcare costs has been quoted to be U.S. $3.9 billion to U.S. 12.5 billion per year^2^. Based on the clinical phenotypes such as the presence or absence of nasal polyps (NPs), CRS can be classified as CRSwNP (with NPs) and CRSsNP (without NPs). Although CRSwNP accounts for only 20–33% of CRS cases^3^, it is associated with significant morbidity and decreases the quality of life, making this disease clinically important to identify, evaluate, and treat^4,5^.

Recently, emerging evidence has suggested that the clinical “phenotypes” do not provide full insight into the underlying cellular and molecular pathophysiologic mechanisms of CRS^6^. In contrast, “endotyping” provides a more comprehensive approach to classify CRS variants and will lead to more precise strategies for specific therapeutic interventions. To update, the endotype for CRSsNP is characterized by a distinct inflammatory pattern with a T helper cell 1 (Th1) cell bias and excess interferon-γ (IFN-γ) and neutrophilic inflammation, whereas CRSwNP in Caucasians is characterized by eosinophilic inflammation, a low level of TGF-β signaling, eosinophil infiltration, and a deficit in Treg cells^7^. CRSsNP is typically linked with an elevation of Th1-associated cytokines and CRSwNP is associated with a Th2 or mixed Th1/Th2 response^8^, though it is now commonly accepted that both CRS can also present with a combination of Th1-, Th2-, and Th17-associated signatures^9^. The effector T-cell differentiation (Th1, Th2, Th17, and Treg), the expression of transcription factors (TFs), effector cytokines, chemokine receptors, and T-cell functions, represent the T-cell subtypes. For example, Th1 cells primarily secret TNF and IFN-γ, whereas Th2 cells release IL-4, 5, 13, etc., and Th17 cells secret IL-6, TNF, and IL-17^10,11^.

Our previous study using a PCR microarray analysis to catalog oxidative stress-related gene expression revealed that the *prostaglandin-endoperoxide synthase 2 (PTGS2)* gene, also commonly named *cyclooxygenase-2 (COX-2)* gene, out of 84 genes is significantly upregulated for 2.31 folds in NP tissues of Taiwanese patients with CRSwNP than nasal mucosa tissues of healthy controls^12^. In this aspect, several studies are showing that COX-2 is inversely downregulated^13,14^ in NP tissues and nasal epithelial cells of NP tissues of Caucasian NP patients^15^. A later study from Gosepath and his colleagues indicates that while COX-1 is strongly up-regulated, COX-2 expression is significantly lower in epithelial cells of NPs than in those of chronic sinusitis without polyps^16^. On the other hand, there is a study showing that eosinophilic NPs are characterized by stronger expressions of COX-2 in glandular and stromal cells but neutrophilic polyps showed significant expressions of COX-2 in epithelial and endothelial cells (ECs). The authors concluded that irrespective of the cellular type, the intensity of expressions in eosinophilic and neutrophilic polyps is significantly higher than in the normal mucosa^17^. Moreover, increased protein expressions of COX-2, p38MAPK, ERK, and NF-κB subunits are detected in epithelial and inflammatory cells and their mRNA levels are significantly higher in CRSwNP than in control tissues^18^. Therefore, the expression level regarding COX-2 in CRSwNP seems to be controversial and may exist in geographical variability which has distinct endotypes in worldwide CRS populations.

Prostaglandins (PGs) are a group of C20 lipid mediators (eicosanoids) synthesized from arachidonic acid (AA) by COX as a key enzyme. The AA-COX pathway produces five bioactive metabolites consisting of four kinds of PGs and thromboxane (TX): PGE_2_, PGD_2_, PGF_2_, PGI_2_, and TXA_2_^19^. It is well established that COX-1 is constitutively expressed, whereas COX-2 can be upregulated in disease conditions. PGE_2_ maintains a local constant and regulates a wide range of physiological activities, including immune responses. In the respiratory system, PGE_2_ can mediate bronchodilation and anti-inflammatory effects via EP2 and/or EP4 receptors^20^. In a pathological condition, PGE_2_ is increased in lung fibroblasts derived from chronic obstructive pulmonary disease (COPD)^21^ and contributes as a mediator of proinflammation and angiogenesis within the airways of COPD subjects through the production of interleukin (IL)-8 and vascular endothelial growth factor^22^. Therefore, PGE_2_ can also be a proinflammatory mediator in respiratory diseases and pathologies^20,23^. It has been reported that the expression of E_2_-prostanoid receptors in NP tissues of smoking and nonsmoking patients with CRS^24^. In addition to PGE_2_, TXB_2_, a stable metabolite of TXA_2_, and PGD_2_ have been recovered from the nasal lavage fluid of patients with allergic rhinitis after allergen provocation^25,26^. Immunohistochemical studies of the nasal mucosa revealed that the TP receptor, a receptor for TXA_2_, is expressed in vascular smooth muscle cells, vascular endothelial cells, epithelial cells, and submucosal glands in human nasal mucosa^27^. Our previous study has also shown abundant TP receptor expression in human CRSsNP mucosae and TXA_2_ mediates CXCL-1 and -8 chemokine upregulation in CRSsNP nasal mucosa-derived fibroblasts^28^.

Given that the significant upregulation of *the COX-2* gene is observed in Taiwanese CRSwNP patients^12^ but a controversial result is observed in a worldwide population, this study sought to investigate the expression, location, and distribution of COX-2 protein in the NP specimens of CRSwNP patients and to confirm whether the corresponding COX-2 protein is upregulated in parallel. Furthermore, NP-derived primary cultured fibroblast cell was established and used to clarify the importance and roles of COX-2 upregulation in the pathophysiology of CRSwNP. It was found a significant increase in COX protein in NP tissues of CRSwNP than that in controls. Moreover, a variety of stimuli related to CRS pathogenesis were capable of causing COX-2 induction and PGE_2_ and TXA_2_ production, and IL-6 cytokine secretion in NP-derived nasal fibroblasts. The peptidoglycan (PGN), a major cell wall component of Gram (+) bacteria, was chosen as a main stimulant to explore the underlying mechanism in causing PGs and IL-6 release in nasal fibroblasts. We demonstrated that COX-2 protein and IL-6 are overexpressed in human NP tissues. At least in response to PGN challenge, the PI3K/Akt activation and its downstream COX-2-mediated PGE_2_ autacoid correlate with IL-6 protein trafficking/production into extracellular space in human NP-derived fibroblasts.

## Results

### Confirmation of a higher COX-2 expression level in NP tissues

To confirm our PCR array result which *COX-2* gene is significantly upregulated for 2.31 folds in NP tissues of Taiwanese patients with CRSwNP than nasal mucosa tissues of controls^12^, the 24 control, CRSsNP, and CRSwNP patients were recruited in this study. The demographic analysis of control and CRS patients was summarized in Supplementary Table 1. A statistical significance was observed in the Lund-Mackay score by CT findings between CRSsNP and CRSwNP. Moreover, the SNOT-22 score, evaluating the severity of complaints that patients have been experiencing over the past weeks due to CRS, was statistically significant among control, CRSsNP, and CRSwNP patients. Finally, the NP score indicated the severity of nasal polyposis.

**Figure 1:**
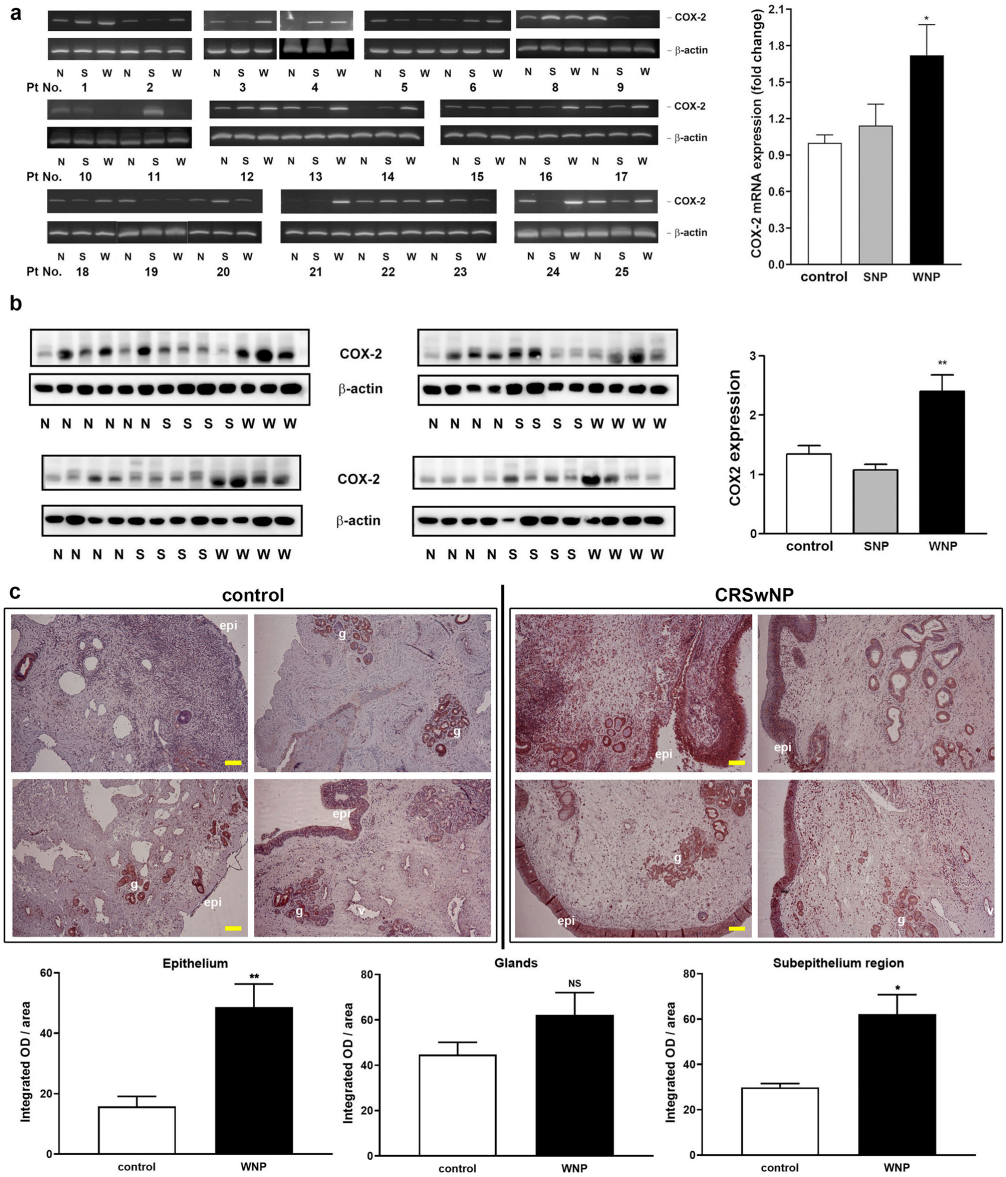
The COX-2 mRNA and protein expression in control and CRSsNP nasal mucosa and CRSwNP NP tissues. (**a**) RT-PCR analysis of human nasal samples. The 24 human control and CRSsNP nasal mucosae and CRSwNP NPs were extracted for preparing total RNA. The total RNAs were analyzed by two-step RT-PCR analysis. The “Pt No.” indicates patient number assigned for this study. Patient 7 was missing due to running out of tissue samples. (**b**) Western blot analysis of human nasal samples. The expression of COX-2 in lysates of the indicated human control and CRSsNP nasal mucosae and CRSwNP NPs were analyzed by Western blotting. N: control; S/SNP: CRSsNP; W/WNP: CRSwNP. *p < 0.05 and **p < 0.01 versus control by Kruskal–Wallis test. (**c**) IHC analysis of COX-2 expression in control nasal mucosa and CRSwNP NP tissues (n = 6). *epi* epithelium; *v* blood vessels, *g* submucosal glands. The quantitation were done by the image analysis software and the mean intensity of epithelium, gland, and subepithelial stroma regions was calculated as the integrated optical density (OD) of total area sizes (pixel square). Yellow-color scale bar = 100 μM.

The COX-2 mRNA level in the control nasal mucosae and CRSwNP NP tissues as well as CRSsNP nasal mucosae were first analyzed by RT-PCR. In total, 24 nasal tissue samples of control, CRSsNP, and CRSwNP patients were measured, it was found that the COX-2 mRNA level in the NP tissues of CRSwNP was significantly higher than that expressed in the control nasal mucosa tissues, as quantified by the densitometric analysis (Fig. 1a). Next, the COX-2 protein expression was examined in nasal tissues of 18 control, 16 CRSsNP, and 15 CRSwNP patients by Western blotting. In Fig. 1b, the relative expression levels of COX-2 protein in control, CRSsNP, and CRSwNP nasal tissues were compared to each other. Among them, 11 out of 18 control patients (61.1%) and 15 of 16 CRSsNP patients (93.5%) were expressed at a relatively low level. However, only 4 of 15 CRSwNP patients (26.7%) were expressed in a low level; i.e. 11 out of 15 (73.3%) were found with a relatively higher expression in the NP tissue samples of CRSwNP patients. The statistical analysis demonstrated that COX-2 protein is significantly expressed at a higher level in NPs of CRSwNP, but not in nasal mucosae of control and CRSsNP.

To further examine the COX-2 expression, location, and distribution in control nasal mucosa and CRSwNP NP tissues, the IHC was then performed. In Fig. 1c, overall, the positive staining in NP tissues was more intensive than that in control nasal mucosa. In the control group, a noted amount of COX-2 expression was observed mainly in subcutaneous glands (gland; g) and partially in the epithelium (epithelium; epi) and vessels (denoted as v); however, of the patients with CRSwNP examined, the COX-2 was markedly increased in the epidermal cell layer and submucosal stroma area. The quantitative analysis revealed a significant increase in COX-2 expression in epithelium and subepithelial stroma regions.

It is known fibroblasts as a major cell type in the subepithelial stroma of tissues. Thus, to investigate the possible sources and regulatory mechanism of COX-2 production in NPs of CRSwNP, the NP-derived fibroblast cells were isolated and prepared from NP tissues and characterized by the immunofluorescence (IF) assay. As shown in Supplementary Fig. 1a, the IF analysis revealed that the isolated cells were highly expressed with vimentin as they were positively stained by the anti-vimentin Ab, but not by the corresponding nonimmune IgG. In parallel, the DAPI staining indicated the location of each cell nucleus with blue fluorescence. Merging of the green fluorescence and DAPI-stained images revealed a strong green fluorescence within the cytoplasm and blue fluorescence within the nucleus (Supplementary Fig. 1b, upper panels). On the other hand, negative staining was observed for the staining of α-SMA (alpha-smooth muscle actin) in cells, a specific marker of smooth muscle cells (lower panels). Therefore, the isolated cells are characterized as fibroblasts but not smooth muscle cells or myofibroblasts.

### The COX-2 is upregulated in nasal fibroblasts challenged with various proinflammatory stimulants

**Figure 2:**
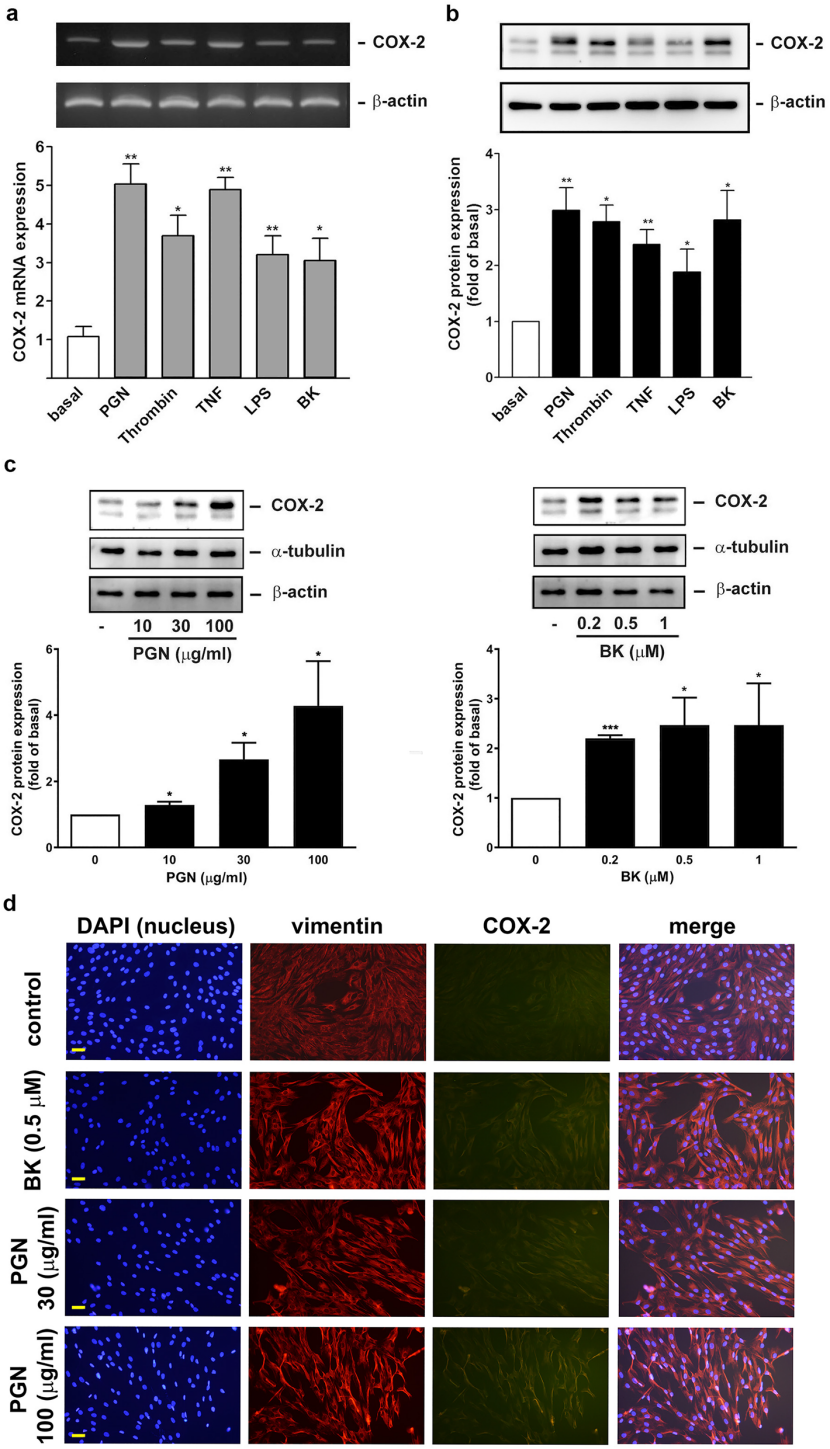
PGN and BK as representatives to induce COX-2 mRNA and protein expression in fibroblasts. (**a**,**b**) The cultured human nasal fibroblasts were stimulated by the indicated substances for (**a**) 6 h or (**b**) 16 h. Cells were collected and prepared and the COX-2 expression was analyzed immediately by (**a**) RT-PCR (n = 4) and (**b**) Western blotting (n = 5) and quantified by densitometry. *p < 0.05 and **p < 0.01 versus control by Kruskal–Wallis test. (**c**) The cultured human nasal fibroblasts were treated with the indicated concentrations of PGN or BK for 16 h, cells were collected and the COX-2 expression was analyzed by Western blotting and densitometry (n = 4). *p < 0.05, **p < 0.01, and ***p < 0.001 versus control. (**d**) The cultured human nasal fibroblasts were treated with the indicated concentrations of PGN or BK for 16 h. The COX-2 and vimentin expression, nucleus (DAPI staining), and their colocalization (merge) were assayed by IF analysis (n = 3).

The characterized NP-derived nasal fibroblasts were further used as a cell model to explore whether inflammatory substances that are involved in CRS pathogenesis can cause an increase in COX-2 expression. These include bacteria cell wall components such as peptidoglycan (PGN) and lipopolysaccharide (LPS), thrombin, tumor necrosis factor-α (TNF-α), and bradykinin (BK). The β-actin protein was taken as the internal control for ensuring the equal loading of total proteins. In Fig. 2a,b, both the RT-PCR and Western blot analyses show that PGN, thrombin, TNF-α, and BK had an inductive effect on COX-2 mRNA and protein expression, with statistically significant differences compared with the control. Surprisingly, LPS at this concentration was found to be a relatively weak inducer of COX-2 mRNA and protein induction in NP-derived nasal fibroblasts.

It was reported that nasal colonization of Gram (+) bacteria- *Staphylococcus aureus* is detected in 67% of patients with CRSwNP^31^ and PGN is a major cell wall component of G(+) bacteria. Moreover, immunohistochemical (IHC) studies of normal and allergic nasal mucosa and several cell types showed immunoreactivity for both B1 and B2 receptors for bradykinin (BK)^32^. Since the cognate receptors, namely Toll-like receptor (TLR) and BK receptor^33^, are responsible for PGN and BK, respectively, and are with distinct properties, PGN and BK were selected as two representatives for the following assays. It was found that PGN and BK significantly caused COX-2 protein induction in a concentration-dependent manner (Fig. 2c). Furthermore, the promoting effect of PGN and BK on COX-2 in nasal fibroblasts was confirmed by the IF microscopic analysis of cells with anti-vimentin and COX-2 antibody and DAPI staining (for identifying cell nucleus), in which vimentin was constitutively expressed and COX-2 upregulation in cytosol was observed in BK- and PGN-treated cells. The overlay (merge) of nucleus, vimentin, and COX-2 images indicated an increase in colocalization of vimentin and COX-2 (orange color) within cytosol in BK- and PGN-treated cells (Fig. 2d).

### Association of COX-2 induction with PGE_2_ and EP4 receptor in IL-6 secretion

**Figure 3:**
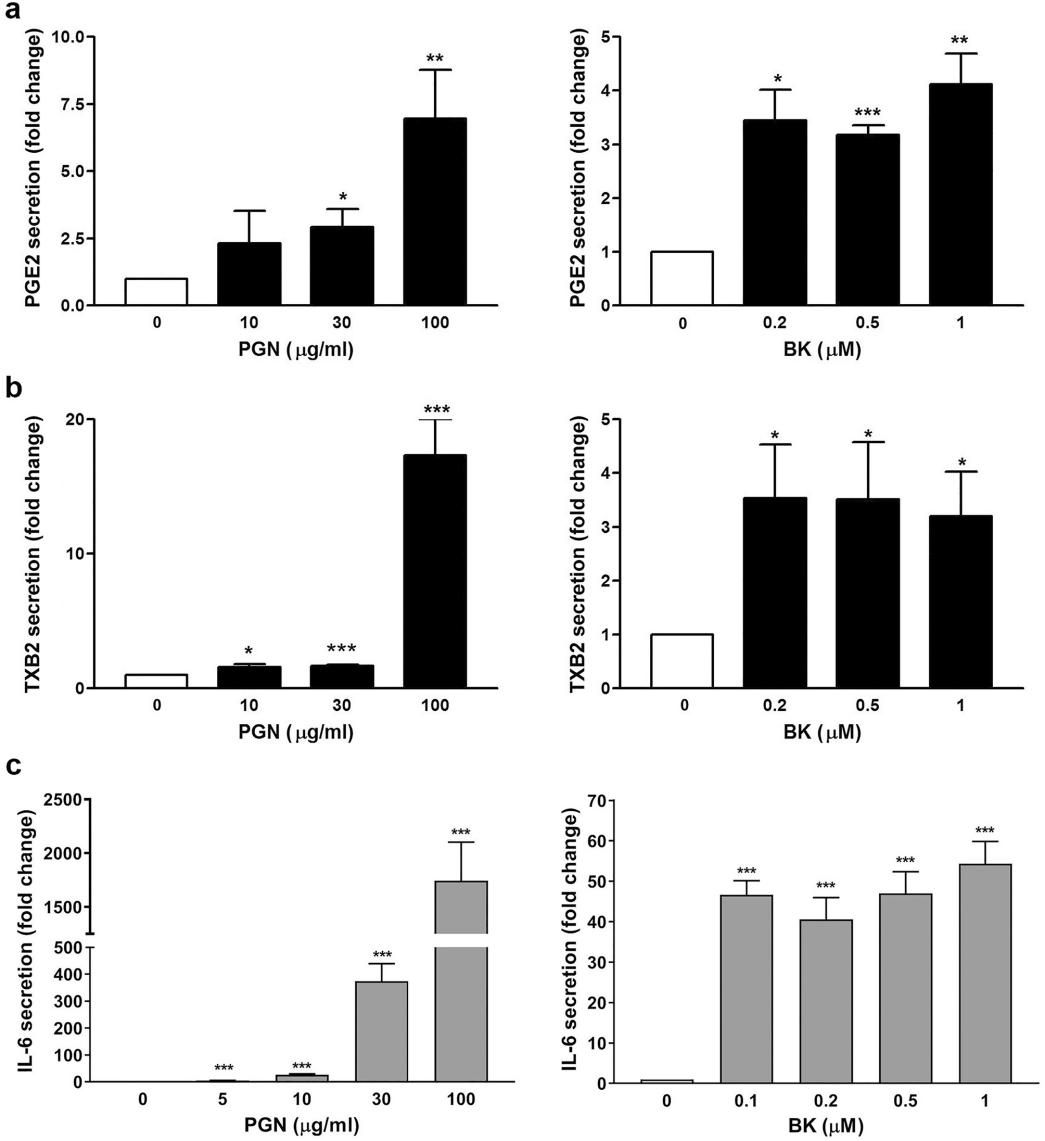
Effect of PGN and BK on PGE_2_ and TXB_2_ production and IL-6 secretion in fibroblasts. The cultured human nasal fibroblasts were treated with the indicated concentrations of PGN or BK for 16 h, the culture media were collected and the (**a**) PGE_2_, (**b**) TXB_2_, and (**c**) IL-6 secretion was determined by ELISA (n = 3). *p < 0.05, **p < 0.01, and ***p < 0.001 versus control.

Past studies have shown that the increase in COX-2 expression is related to the release of interleukin-6 (IL-6) through PGE_2_ production^29,30^. Therefore, the PGE_2_ and TXB_2_ (a stable metabolite of TXA_2_) and IL-6 secretion were analyzed by ELISA. In Fig. 3a,b, PGN and BK treatment significantly induced PGE_2_ and TXB_2_ production in the fibroblast culture medium. The PGN provoked a robust increase in PGE_2_ and TXB_2_ at a concentration of 100 μg/ml, whereas the induction for both PGs by BK appeared not to be in an entirely concentration-dependent fashion. Next, the ability of PGN and BK to cause IL-6 generation was investigated. In accordance with the findings presented in Fig. 3a,b, PGN and BK also significantly induced an increase in IL-6 secretion into the culture medium (Fig. 3c).

**Figure 4:**
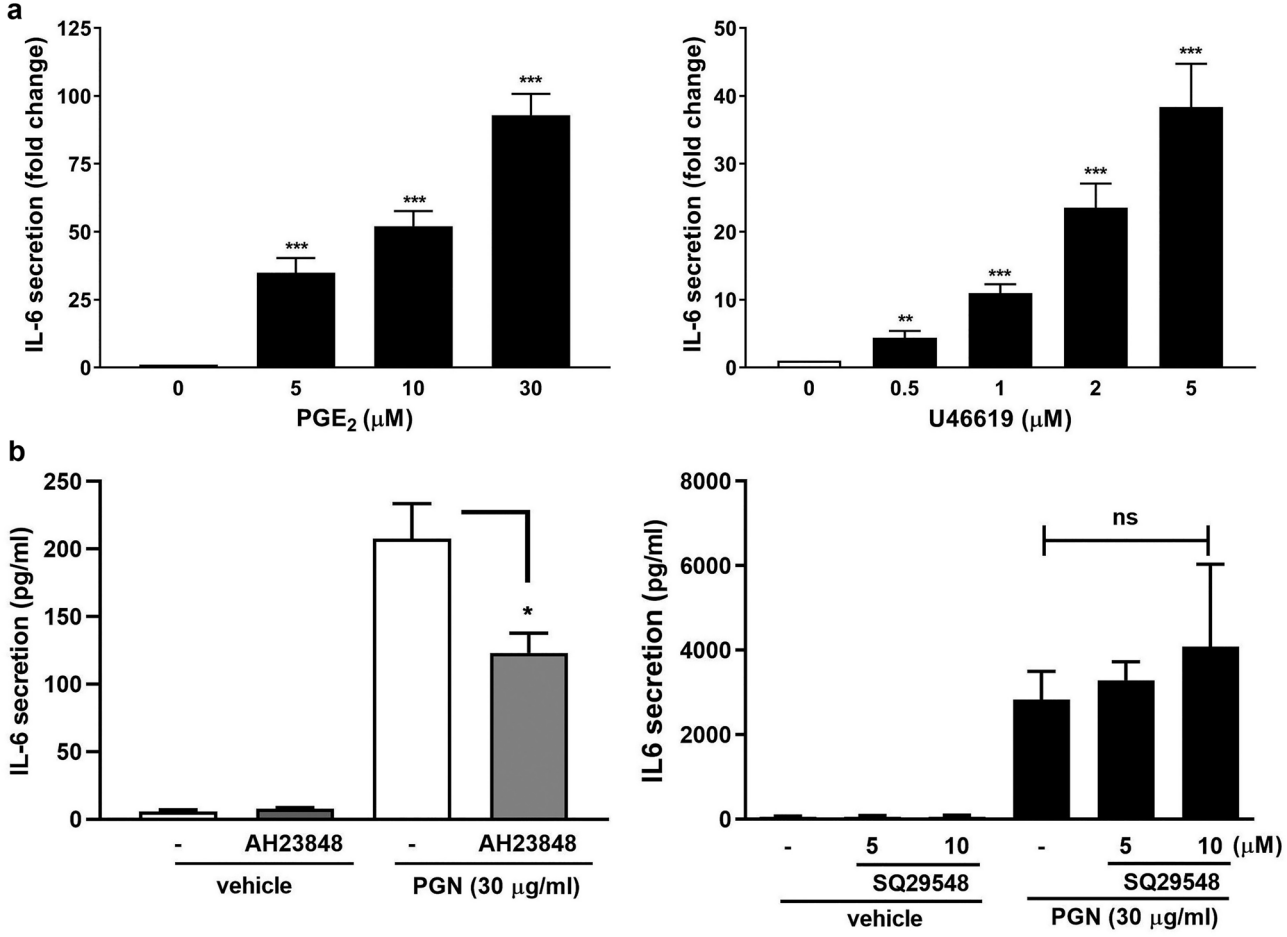
Requirement of PGE_2_ and EP_4_ receptor in PGN-induced IL-6 secretion. The human nasal fibroblasts were challenged with the indicated concentration(s) of (**a**) PGE_2_ or U46619 (a TXA_2_ analog) for 16 h, the cell culture media were collected and the IL-6 secretion was analyzed by ELISA. The results were performed in triplicates for each experiment (n = 3). ***p < 0.001 versus control. (**b**) The human nasal fibroblasts were challenged with PGN (30 μg/ml) in the presence or absence of the prostanoid receptor antagonist-AH23848 (10 μM) or SQ29548 (10 μM) for 16 h. At the end of incubation, the cell culture media were collected and the IL-6 secretion was analyzed by ELISA (n = 3–4). NS: non-significant. *p < 0.05, **p < 0.01, and ***p < 0.001 versus control.

Since PGE_2_ and TXA_2_ have been known to be members of the autacoid family^34^, it is hypothesized that they might act as an autacoid to activate their respective receptors to induce IL-6 secretion. To identify which of the secreted PGE_2_ and TXA_2_ is associated with IL-6 secretion, the nasal fibroblasts were treated with PGE_2_ or U46619 (a TXA_2_ analog). It was found that both PGE_2_ and U46619 were able to enhance IL-6 secretion in a concentration-dependent manner (Fig. 4a). The AH6809, an EP and DP receptor antagonist with nearly equal affinity for the cloned human EP1, EP2, EP3-III, and DP1 receptors, did not exhibit any inhibition toward PGN-induced IL-6 secretion (Supplementary Fig. 2). However, AH 23848, a dual antagonist toward TP and EP_4_ receptors, but not the TP antagonist, SQ29548, exhibited a significant inhibition toward PGN-induced IL-6 secretion (Fig. 4b). The results suggest that PGE_2_ and EP_4_ receptor are essential for PGN-induced IL-6 secretion.

### Requirement of PI3K/Akt pathway and COX-2 induction for IL-6 secretion

**Figure 5:**
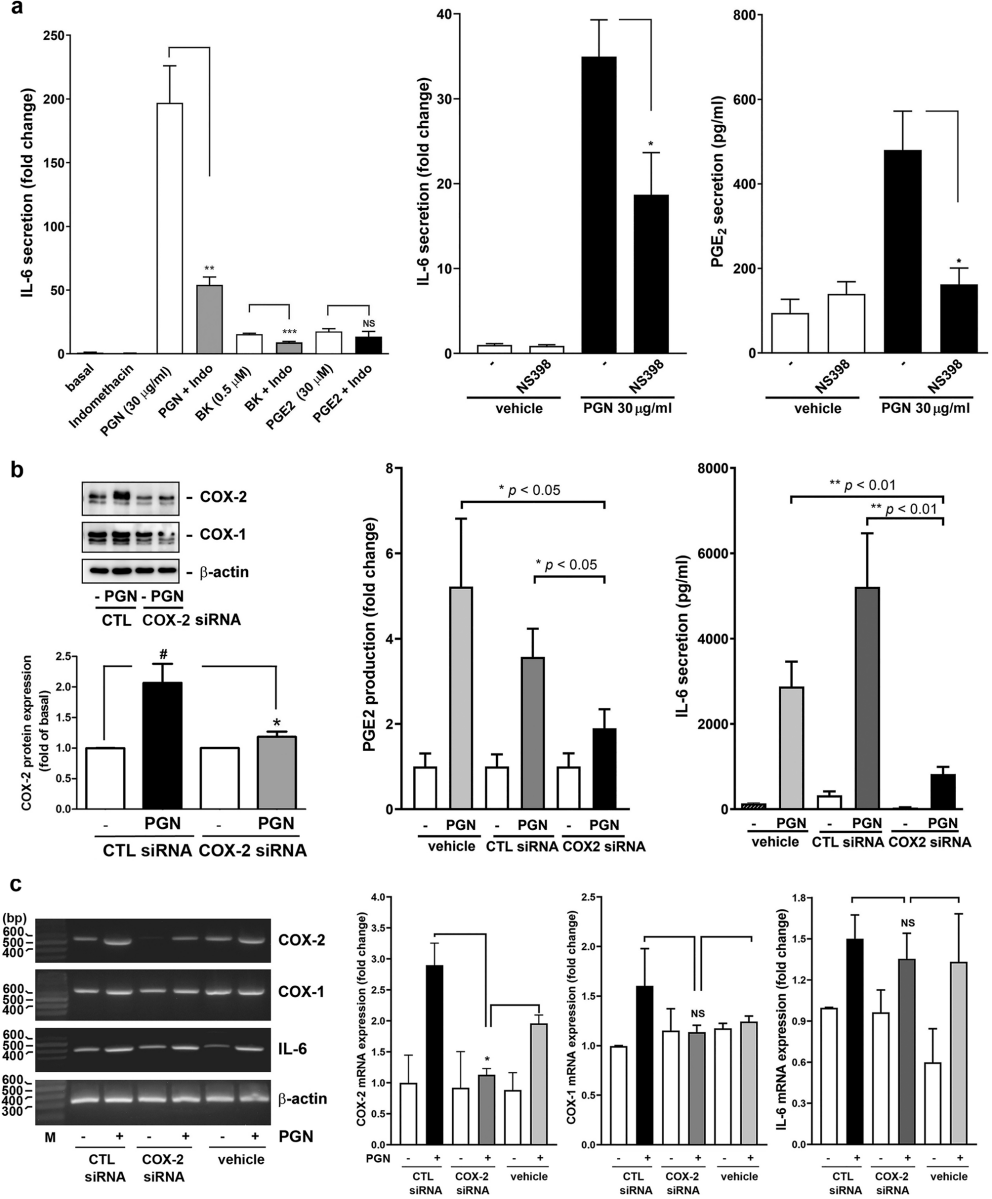
The inhibition and KD of COX-2 compromise PGN-induced PGE_2_ and IL-6 production/secretion. (**a**) The human nasal fibroblasts were challenged with PGN, BK, or PGE_2_ in the presence or absence of indomethacin (COX-1/-2 inhibitor) or NS398 (COX-2 inhibitor) for 16 h. At the end of incubation, the cell culture media were collected and the IL-6 and PGE_2_ secretion was analyzed by ELISA (n = 3). (**b** and **c**) The human nasal fibroblasts were transfected with control (CTL) or COX-2 siRNA for 72 h and followed by stimulation with vehicle or PGN for (**b**) 16 h or (**c**) 6 h. (**b**) The specific KD effect on COX-2 protein expression was determined by Western blotting and densitometry (n = 3), whereas the PGE_2_ and IL-6 secretion in culture media were determined by ELISA (n = 3). (**c**) The effect of COX-2 KD on PGN-induced COX-2, COX-1, and IL-6 mRNA expression was determined by RT-PCR and densitometry (n = 3). NS: non-significant. ^#^*p* < 0.05 versus control basal. *p < 0.05, **p < 0.01, and ***p < 0.001 versus non-transfected control or control siRNA-treated group.

Then, it is of concern whether COX-2 upregulation is directly associated with the increase of PGE_2_ and IL-6 secretion in the culture medium. To clarify this question, a pharmacological intervention was performed. As shown in Fig. 5a, the treatment of nasal fibroblasts with a nonselective clinically-used COX-1 and COX-2 inhibitor, indomethacin, markedly and significantly attenuated PGN- and BK-induced IL-6 production, as determined by ELISA. However, it did not interfere with PGE_2_-induced IL-6 expression, indicating that PGN and BK, but not PGE_2_, induce IL-6 expression through COX-1 and/or COX-2 (left panel). Moreover, NS-398, a selective COX-2 inhibitor, compromised PGN-induced IL-6 secretion and PGE_2_ production even at a very low concentration of 1 nM (middle and right panels). Taken together, these implied a requirement of COX-2 induction for IL-6 secretion.

To confirm the above speculation, the siRNA interference was introduced to nasal fibroblasts to knock down (KD) COX-2 mRNA and protein expression to investigate whether the corresponding IL-6 release is compromised in parallel. As shown in Fig. 5b, it was found that the COX-2 KD significantly reduced COX-2 but not COX-1 protein expression. Under this condition, the PGN-induced PGE_2_ and IL-6 secretion were significantly reduced in the culture medium, as assayed by ELISA. However, specifically knocking down PGN-induced COX-2 mRNA did not affect the PGN-provoked IL-6 mRNA transcriptional level (Fig. 5c). Consistently, treatment of cells with the COX-2 inhibitor-NS398 also did not affect PGN-provoked IL-6 mRNA expression (Supplementary Fig. 3).

**Figure 6:**
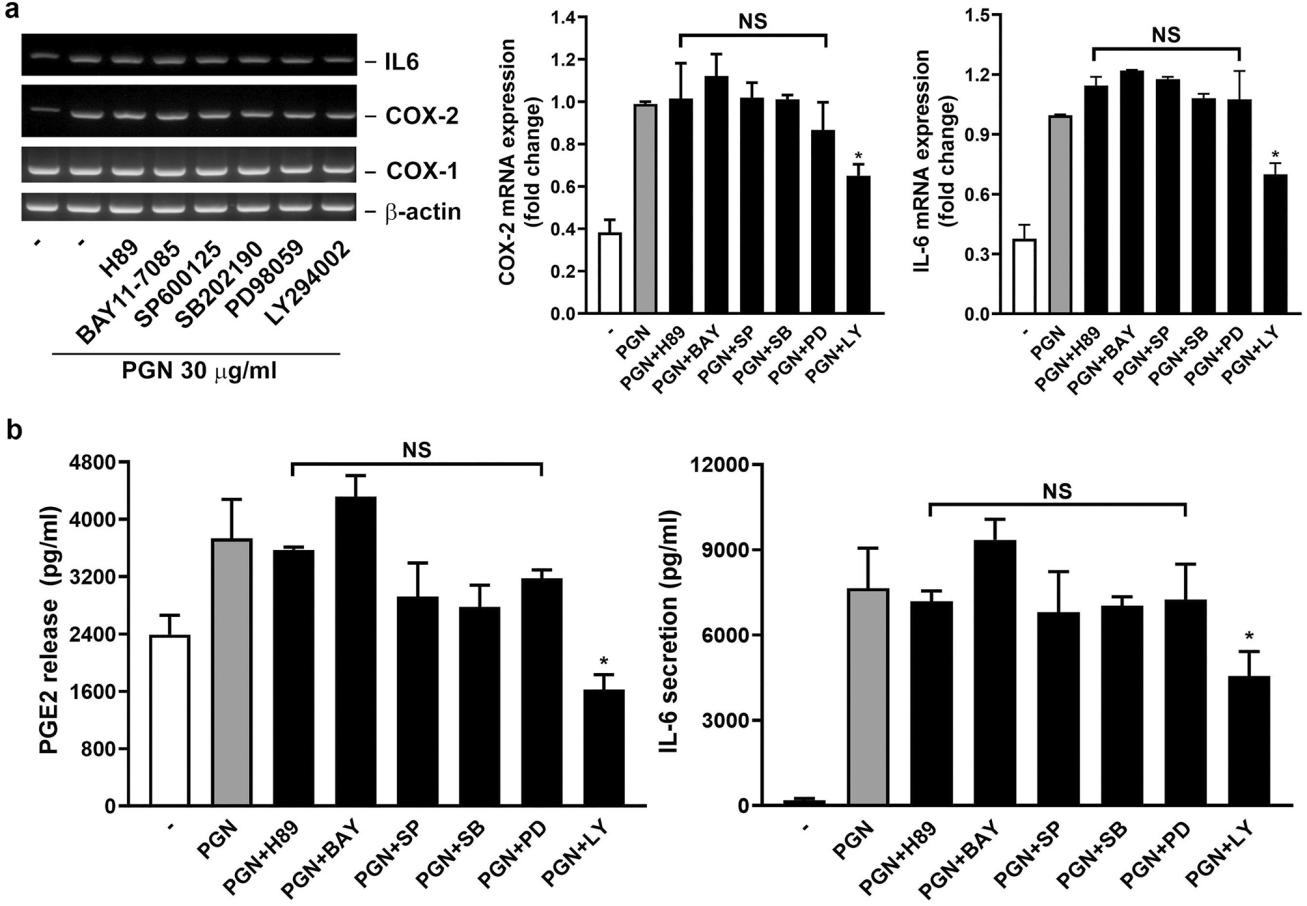
The inhibition of PI3K/Akt attenuates PGN-induced COX-2 and IL-6 mRNA expression and PGE_2_ and IL-6 secretion. The cultured human nasal fibroblasts were stimulated with PGN in the presence of vehicle (-) or the indicated cellular signaling inhibitor for 6 h. (**a**) Cells were collected and the COX-2, COX-1, IL-6, and β-actin mRNA expression were analyzed immediately by RT-PCR, whereas (**b**) the PGE_2_ and IL-6 level in culture media were analyzed by ELISA. SP (SP600125), SB (SB203580), PD (PD098059), and LY (LY294002): 10 μM; H-89 and BAY (BAY 11–7082): 5 μM. The quantitation of mRNA expression level was performed by densitometry (n = 3). *p < 0.05 compared with PGN control by Kruskal–Wallis test. *NS* non-significant.

Next, to further identify the possible upstream cellular component(s) responsible for COX-2 and IL-6 induction, an RT-PCR analysis was performed in cells treated with PGN in the presence of various cellular signaling inhibitors. The following pharmacological signaling inhibitors were used, including H-89 for protein kinase A (PKA), BAY 11-7082 (BAY) for NF-κB/IκB pathway, SP600125 (SP) for JNK, SB203580 (SB) for p38 MAPK, PD098059 (PD) for MAPKK, and LY294002 (LY) for PI3K/Akt pathway. Surprisingly, only the blockade of the PI3K/Akt pathway by LY294002 significantly compromised the PGN-induced COX-2 and IL-6 mRNA induction. In the meantime, the PGE_2_ release and IL-6 secretion were also reduced in the presence of LY294002 (Fig. 6). These suggest that PI3K/Akt activation is not only required for PGN-induced COX-2 mRNA transcription, PGE_2_ release, and IL-6 secretion, but may also be needed for direct driving IL-6 mRNA transcription.

### Involvement of nasal fibroblasts in Th17-related cytokine production

**Figure 7:**
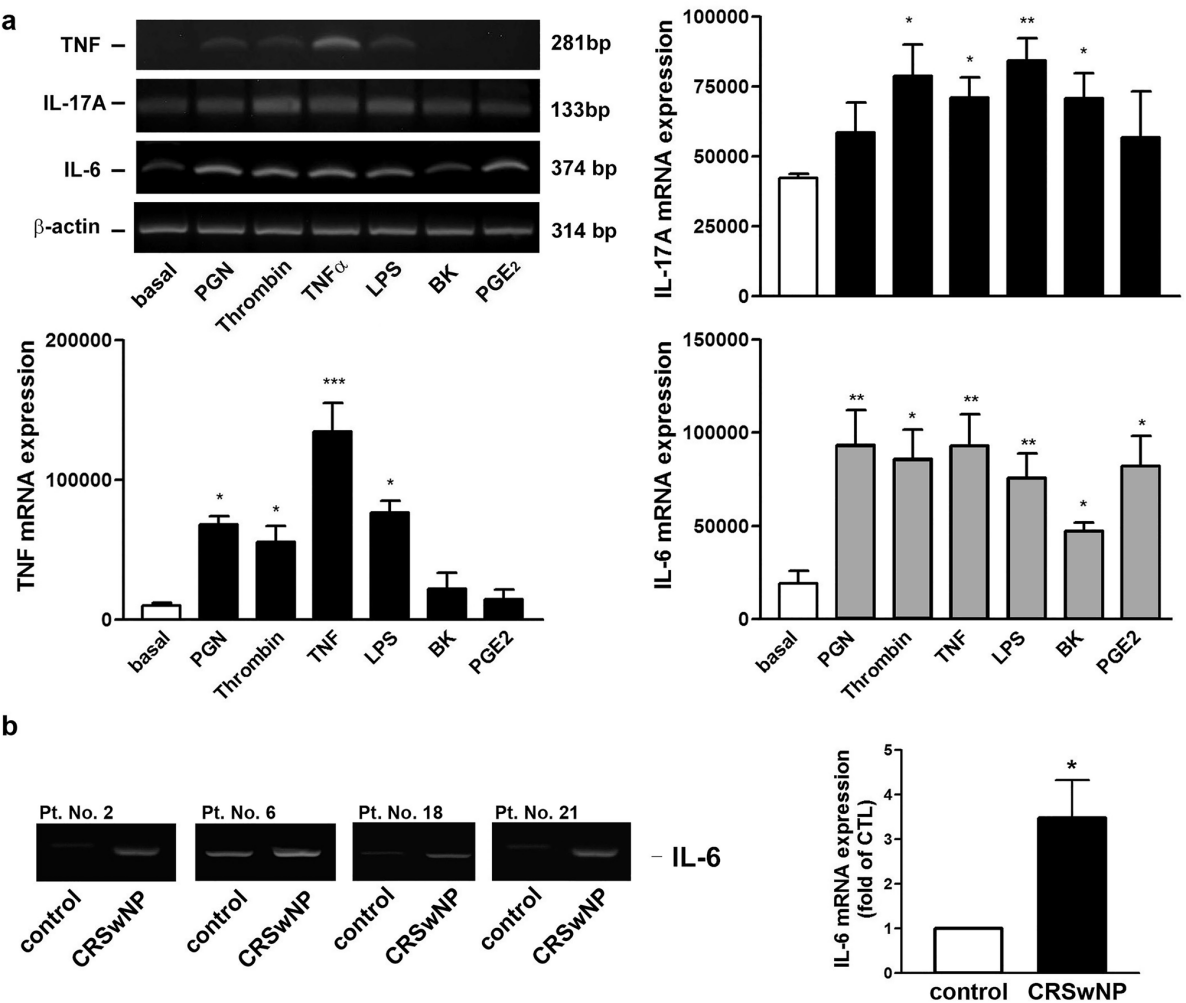
Role of the CRS-related stimulants in Th17-related cytokine production in nasal fibroblasts and IL-6 expression in NP tissues of CRSwNP. (**a**) The human nasal fibroblasts were treated with a vehicle or the indicated stimulator for 6 h. Cells were collected and the Th17-related cytokine mRNA expression was analyzed by RT-PCR and quantified by densitometry (n = 4). This was representative of 4 independent experiments. *p < 0.05, **p < 0.01 and ***p < 0.001 compared with basal by Kruskal–Wallis test. (**b**) The randomly selected cDNAs from 4 control and CRSwNP nasal tissues in Fig. 1 were analyzed by PCR for examining the IL-6 mRNA expression levels. The β-actin expression in each corresponding patient was shown in Fig. 1A and the results were quantified by densitometry (n = 4).

As described earlier, it is now commonly accepted that both CRS can also present with a combination of Th1-, Th2-, and Th17-associated signatures^9^. Since Th17 cells mainly secret IL-6, TNF, and IL-17^10,11^, we tested whether the various stimulators linking to CRS pathophysiology that can induce COX-2 expression are also associated with the production of these cytokines in NP-derived fibroblasts through transcriptional regulation. Therefore, the RT-PCR was performed. As shown in Fig. 7a, PGN, thrombin, TNF, LPS, and BK differentially caused TNF, IL-17A, and IL-6 mRNA expression in nasal fibroblasts. However, PGE_2_ could only induce IL-6 expression. Therefore, although some Th17-related cytokines could be produced in nasal fibroblasts in response to various stimuli, the COX-2-driven PGE_2_ release is only associated with IL-6 production.

Finally, we examined whether IL-6 induction can be found in NP tissues of CRSwNP, the random 4 tissue samples of control and CRSwNP were selected and their same cDNAs used in Fig. 1a were reanalyzed by PCR using IL-6 primers for their expression levels of IL-6 mRNA. As shown in Fig. 7b, IL-6 mRNA was significantly increased in NP tissues of CRSwNP, revealing an increase in IL-6 in nasal polyposis. The even expression of β-actin in the control and patients could be found in Fig. 1a.

## Discussion

Recent studies showed that the classification of CRS based on “inflammatory endotypes” (i.e. pathophysiology) may be a better way to develop better treatments. Meanwhile, many otolaryngologists suggest that in addition to inflammatory endotyping, other biomarkers that identify relevant endotypes will be needed. However, while evidence-based medicine is recommended in the medical area, it is of concern which evidence should be based on the properties and endotypes of some diseases that may vary among worldwide populations. In this study, we observed that COX-2 mRNA and protein were significantly expressed at a higher level in the NP tissues of Taiwanese CRSwNP patients, as assayed by RT-PCR and Western blotting. Moreover, the COX-2 was mostly presented in the epithelium layer, submucosal glands, and stroma in the NP tissues (Fig. 1). These results confirm our previous findings on higher COX-2 gene expression via the real-time PCR array^12^. In contrast to some previous studies having controversial results on COX-2^13–16,18^. We can assure here that COX-2 mRNA and protein are upregulated in NPs of Taiwanese CRSwNP. Since we have obtained human clinical nasal tissue samples from patients and demonstrated the roles of COX-2 expression both in human nasal polyposis and NP-derived fibroblasts. Therefore, we did not further use the NP murine model for experiments aimed at studying the effects of COX-2.

Regarding the upregulation of COX-2 in NP tissues, a study has shown that an increase of the COX-2 gene expression can be linked to the development of NPs in patients with CRS and the -14C/G MET gene polymorphism and -765G/C genotype of COX-2 can be associated with an increased risk of the occurrence of CRS with NPs in the Polish population^35,36^. Recently, it was reported that the mucin 5AC-enriched exosomes could be effectively taken up by CRSsNP-derived fibroblasts, resulting in a significant increase in the expression of COX-2, VEGF, and MMP-9^37^. In addition, the research aimed to analyze the expression of transcript variants of *PTGS1* *(COX-1)* and *PTGS2* (*COX-2*) genes in the pathobiology of CRSwNP indicates that the COX1.3, COX1.4, COX1.5, and COX2.1 variants may promote milder CRSwNP course. On the contrary, the variants COX1.1, COX1.2, and COX2.2 may be involved in a more aggressive CRSwNP disease^38^. Therefore, our current results not only provide information concerning COX-2 expression and distribution in NP tissues but may also suggest the existence of geographic variability among human species with CRSwNP.

COX-2 is a well-known gene product that is necessary for mediating PG synthesis. On the other hand, COX-2 is also a ROS-related gene product associated with neovascularization^39,40^. PTGS2 (COX-2) expression contributes to the generation of oxidative stress such as promoting PGE_2_ and NOX1 and 4 and inhibiting heme oxygenase-1 expression^41^. In this study, we observed a robust increase in COX-2 expression associated with IL-6 secretion in nasal fibroblasts’ response to various stimuli related to CRS pathogenesis (Figs. 2 and 5). Moreover, these various stimuli could provoke expression in Th17-related cytokines such as IL-6, TNF, and IL-17 in NP-derived nasal fibroblasts (Fig. 7a) and IL-6 in an *in-vivo* NP tissue (Fig. 7b), suggesting the inflammatory cytokine profiles in CRS might not only result from T cells but also stroma cells like fibroblasts in vivo. Our findings highlight that the treatment of CRSwNP patients in Taiwan or other countries with a similar COX-2 endotype may benefit from using a corticosteroid or a selective COX-2 inhibitor to improve COX-2-mediated inflammation and oxidative stress in nasal polyposis.

The association of COX-2 induction with IL-6 secretion can be evidenced by the following observations. First, indomethacin targeting COX-1/COX-2 and NS-398 preferentially and selectively blocking COX-2 activity compromised PGN- or BK-provoked IL-6 secretion (Fig. 5a). Second, siRNA interference specifically downregulated COX-2 but not COX-1 protein expression led to a substantial decrease in IL-6 secretion (Fig. 5b). It has been reported that cigarette smoke extract- (CSE-) and sphingosine-1-phosphate-enhanced COX-2 expression is related to IL-6 release through PGE_2_ production in human tracheal smooth muscle cells^29,30^. However, in this study, it is surprising that the diminishing of COX-2 expression by siRNA interference and inhibition of COX-2 activity by NS398 did not affect PGN-induced IL-6 mRNA expression level (Fig. 5c and Supplementary Fig. 3), suggesting that COX-2-mediated IL-6 secretion is not regulated via transcription. We speculated that PGN provokes IL-6 mRNA and protein expression through, at least in part, two independent pathways. One is the COX-2-independent and another is the COX-2-dependent pathway. The activation of the COX-2-dependent pathway leads to PGE_2_ autacoid release which can enhance IL-6 secretion in extracellular space possibly via regulating the transportation of IL-6 protein. That’s why siRNA KD of COX-2 resulting in extracellular PGE_2_ reduction is accompanied by a decrease in IL-6 protein secretion but not IL-6 mRNA expression. A similar study also reports that PGN-induced IL-6 production in RAW 264.7 macrophages is mediated by COX-2, EP_2_/EP_4_ receptors, protein kinase A, and NF-κB signaling pathway and in which the authors demonstrate that PGN can induce COX-2-independent and -dependent signaling pathways^42^. However, one of the major differences between the two studies is that the induced PGE_2_ autacoid seems to enhance IL-6 expression by affecting its transportation in this study. In this regard, one may question the speculation proposed by us as the exogenous addition of PGE_2_ (at the μM range) to nasal fibroblasts also caused an increase in IL-6 mRNA (Fig. 7a). We hypothesize that PGE_2_ affects IL-6 depending on its concentration in extracellular space. That is to say, PGE_2_ tends to enhance IL-6 gene transcription at a relatively higher concentration but prefers to affect IL-6 protein transportation at a lower concentration (at the pM-nM range).

Most cellular signaling downstream toll-like receptors (TLRs) involve NF-κB-dependent pathways^43,44^. However, in Fig. 6, NF-κB, ERK1/2, p38 MAPK, and JNK are unlikely to participate in PGN-induced COX-2 induction and PGE_2_ and IL-6 production. One may concern that the ineffectiveness of the above inhibitors resulted from inappropriate concentrations. This is also unlikely as the working concentrations of these inhibitors have been tested and shown to be workable in our previous studies. Since LY294002 could inhibit PGN-induced COX-2 mRNA induction and PGE_2_ and IL-6 production, it is highly suspected that PI3K/Akt is involved in the COX-2 induction; i.e. they are activated upstream COX-2. However, as LY also reduced PGN-induced IL-6 mRNA expression, perhaps PI3K/Akt directly participates in IL-6 mRNA induction. The participation of PI3K/Akt in PGN-mediated signaling has not been extensively reported. It has been shown that PGN induces CXCL8 expression in monocytes/macrophages through TLR2, PI3K/Akt/mTOR, PKC, ROS, and MAPK^45^.

According to previous studies, the cases of CRSwNP seem to have different clinical, histological, and immunological features and treatment outcomes. The eosinophilic CRSwNP responds well to steroid therapy^46,47^; however, noneosinophilic CRSwNP tends to have resistance to steroid therapy, especially if it is neutrophil-dominant^48^. Therefore, the treatment success of CRS will likely depend on appropriate patient selection and an improved understanding of its pathophysiology. That’s why recent studies focus on endotypes and endotype-based therapeutic strategies in CRS, which may have an application in precision medicine or personalized medicine in the near future^49^. This study provides evidence that COX-2 expression-mediated induction of PGE_2_ autacoid correlates with IL-6 production and secretion in *in-vitro* NP-derived fibroblasts and *in-vivo* NP tissues. Moreover, the NP-derived fibroblasts also contribute to Th17-related cytokine production. The signaling pathway required for such IL-6 production is, at least in part, mediated through PGE_2_ but not TXA_2_ production, which acts in an autacoid pathway and is independent of COX-2-mediated IL-6 mRNA transcription. The proposed schematic illustration of the stimuli-COX-2-PGE_2_ axis in causing IL-6 is summarized in Supplementary Fig. 4. Our findings first elucidate the mechanism of COX-2 induction and PGE_2_ production possibly playing a pathological role in nasal polyposis. The study may also highlight the importance of geographic variability in worldwide people/populations. Therefore, it is highly recommended the clinical involvement of corticosteroid and selective COX-2 inhibitors in treating CRSwNP patients in Taiwan and other countries with similar COX-2 endotyping.

## Materials and methods

### Materials

Peptidoglycan (from *Staphylococcus aureus*, lot number 77140), lipopolysaccharide (LPS), thrombin, and bradykinin (BK) were purchased from Sigma-Aldrich Chemical Co. (St Louis, MO, USA). The COX-1 antibody was obtained from Santa Cruz Biotechnology Inc. (Dallas, TX, USA), whereas the COX-2 antibody was purchased from BD Biosciences (Franklin Lakes, NJ). The β-actin antibody was purchased from EMD Millipore (Billerica, MA, USA). U46619 (a TXA_2_ analog), PGE_2_, AH6809, and AH23848 were purchased from Cayman Chemicals (Ann Arbor, Michigan, USA). α-SMA antibody was purchased from GeneTex Inc. (Hsinchu, Taiwan).

### Patient recruitment and sample collection

This research study received approval from the Ethics Committee of the Shin Kong Wu Ho-Su Memorial Hospital, Taipei, Taiwan, under permission (IRB permission No. 20161210R). The study was conducted with the written informed consent of the patients involved. We confirmed that all experiments were performed in accordance with the institutional relevant guidelines and regulations. A total of 24 patients without any nasal diseases (control group) due to nasolacrimal duct obstruction and 24 patients with CRSwNP and CRSsNP were recruited in this study. To diagnose CRSwNP, the criteria from the EPOS 2020 were followed^50^. Patients were required to have two symptoms, one of which must be nasal obstruction and/or discolored discharge ± facial pain/pressure ± reduction or loss of smell lasting for more than 12 weeks. Additionally, patients must show either endoscopic signs of NPs and/or mucopurulent discharge primarily from the middle meatus and/or edema/mucosal obstruction primarily in the middle meatus and/or a CT scan showed mucosal changes within the osteomeatal complex and/or sinuses. The staging of nasal polyposis was based on the Meltzer Clinical Scoring System^51^. CRSsNP was diagnosed based on patient history and the findings from anterior rhinoscopy, nasal endoscopy, and sinus computed tomography. The above control, CRSsNP and CRSwNP patients had not been treated with oral or topical anti-allergic agents or steroids for at least two months. None of the patients had a history of active allergy, asthma, or aspirin sensitivity. Additionally, none of these cases were in an actively infected status, and no antibiotics were given before the surgical procedure. During functional endoscopic sinus surgery (FESS), the nasal mucosa, including the ethmoidal mucosae, the mucosae around the osteomeatal complex, and the NP tissues, were obtained through the procedure of uncinectomy and ethmoidectomy. In the control group, the agger nasi sinus cell mucosae were prepared during dacryocystorhinotomy procedures.

### Preparation of NP-derived primary cultured fibroblast cells

The nasal fibroblasts were derived from NPs of patients with CRSwNP. The preparation was to place the fragments of nasal NP tissues in a 6-well culture dish in DMEM. The DMEM medium was supplemented with 10% fetal bovine serum (FBS), 100 U/ml penicillin, and 100 μg/ml streptomycin and 2 μg/ml amphotericin (fungizone B), cultured in a 37 °C and 5% CO_2_ incubator, the migrated cells were characterized by immunocytochemistry (ICC) with the presence of fibroblast cell marker.

### Immunofluorescence microscopy

Cells were washed, fixed with 1% paraformaldehyde (PAF) for 20 min, and permeabilized with 0.1% Triton X-100 for 10 min. Cells were blocked with 3% BSA, incubated with the Ab specific for vimentin (1:250; Santa Cruz Biotechnology; mouse anti-human type) and COX-2 (1:100; Cell Signaling; rabbit anti-human type) at 4 °C overnight, and then followed by the anti-mouse 2nd FITC- and anti-rabbit 2nd-rhodamine conjugated Ab (Chemicon) for an additional 1 h at RT. By the end of incubation, DAPI (1:1000; Thermo Fisher Scientific) was added to incubation for cell nucleus staining. After a brief wash, the tissue samples mounted on chamber slides were analyzed under a Nikon Eclipse Ti-S fluorescence microscope (Japan) and photographed using a digital camera. The quantitation of the staining results was performed using the Invitrogen Celleste 5.0 Image Analysis Software (Thermo Fisher). The areas of positive staining for control and CRSwNP patients’ regions of interest (ROI) in the captured images were identified by setting a cutoff value and computed according to the software instructions. The mean intensity of each ROI was calculated as the integrated optical density (OD) of all areas/total area sizes (pixel square) in an ROI. The mean intensity of positive staining was obtained by the above intensity/total ROI number.

### RT-PCR analysis for mRNA expression

The two-step RT-PCR was performed, which allows for the ability to convert all the messages in an RNA sample into cDNA and allows for archiving of samples to analyze several genes. The oligonucleotide PCR primers targeting human COX-1 and COX-2, IL-6, and β-actin were synthesized and listed in Table 1. The total RNA extraction, 1^st^ strand cDNA synthesis, PCR, and PCR product analysis were performed as previously described^52^.

**Table 1:** Primers for RT-PCR.

Gene	Forward primer (5′–3′)	Reverse primer (5′–3′)	Product size (bp)
*IL-6*	TTCAATGAGGAGACTTGCC	TGACCAGAAGAAGGAATGC	374
*COX-2*	CAGCAAATCCTTGCTGTTCC	TGGGCAAAGAATGCAAACATC	517
*COX-1*	ACATTCAGTTCCCACCATCT	TCACTGCTGTTGGGTCTCTG	601
*TNF*	AACATCCAACCTTCCCAAACGC	TGGTCTCCAGATTCCAGATGTCAGG	281
*IL-17A*	CGCAATGAGGACCCTGAGAG	GGACCAGGATCTCTTGCTGG	133
*β-actin*	ATCATGTTTGAGACCTTCAA	CATCTCTTGCTCGAAGTCCA	314

### Cell lysate preparation and Western blot analysis

Cell lysates were prepared as previously described^53^. Total proteins were separated by electrophoresis through SDS–polyacrylamide gels, electroblotted onto PVDF membranes, and then probed using a primary antibody (Ab). The immunoblots were developed using Immobilon Western Chemiluminescent HRP Substrate (EMD Millipore Corporation, Billerica, MA, USA). The membranes were stripped with stripping buffer (62.5 mM Tris–HCl, pH 6.7, 2% SDS, and 100 mM β-mercaptoethanol), washed and reprobed with Abs to examine the levels of α-tubulin or the corresponding total proteins, and then developed.

### Immunohistochemical analysis of COX-2 expression in nasal tissues

The COX-2 expression in nasal mucosa tissues of control and NP tissues of CRSwNP was determined through immunohistochemistry (IHC). Briefly, tissue sections were deparaffinized, and the slides were hydrated in graded ethanol before use. The sections were subsequently washed in Tris-buffered saline (TBS, 10 mM Tris–HCl, 150 mM NaCl, pH 7.4) containing 1% CaCl_2_, immersed in sodium citrate buffer (pH 6.0), and heated in a water bath for 20 min. The slides were incubated at 4 °C overnight with the primary Ab specific for COX-2 (Abcam, Cambridge, MA, USA) after blocking with a buffer containing 10% FBS. The slides were then washed with TBS, incubated with Super Enhancer and Poly-HRP, and then developed with one-step 3-amino-9-ethyl carbazole (AEC) using the Super Sensitive Polymer-HRP IHC Detection System (Biogenex Laboratories, Inc., Fremont, CA, USA) for 5–30 min. Sections were counterstained in hematoxylin for 20–40 s, washed with tap water, and mounted with 100% glycerol.

### ELISA measurement of PGs and IL-6 secretion in culture medium

The PGE_2_ and TXB_2_ production in the cell culture medium was determined by the PGE_2_ and TXB_2_ EIA kits, respectively (Cayman Chemical Company, Ann Arbor, MI) according to the manufacturer’s protocol. Briefly, the culture media were collected and centrifuged and PGE_2_ and TXB_2_ secretion were measured by the acetylcholine esterase competitive enzyme immunoassay. The product of this enzymatic reaction was yellowish in color and absorbs strongly at 412 nm. The intensity of this color is proportional to the amount of PGE_2_ and TXB_2_ tracer bound to the well, which is inversely proportional to the amount of free PGE_2_ and TXB_2_ present in the well during the incubation. Meanwhile, the expression level of IL-6 in the culture medium was determined by the human IL-6 DuoSet ELISA development kit (R&D Systems, Inc., MN, USA). After treatment, the culture media were collected, centrifuged to remove cell debris, and the IL-6 expression was measured at 450 nm with a reference wavelength of 570 nm for correction. The absolute concentrations of PGE_2_, TXB_2_, and IL-6 were determined based on their respective standard curves.

### SiRNA interference

SiGenome control and COX-2 siRNAs were purchased from Dharmacon RNAi Technologies (Thermo Fisher Scientific) and a similar transfection assay has been performed by our lab^28^. The cell cultures were transfected with control or COX-2 siRNAs (150 nM) for 72 h using the DharmaFECT transfection reagent. The cells and media were collected for further analysis.

### Statistical analysis

The data are expressed as the mean ± standard error mean (SEM). A comparison of the means of two groups of data was performed by using the unpaired, two-tailed Student’s *t-test*, whereas Kruskal–Wallis test was performed for comparisons among three or more than three groups of data.

### Supplementary Information

Supplementary Information.

## Data Availability

The raw data used to support the findings of this study have been provided in the supplements.
