# Supplementary material for: The cyclooxygenase-2 upregulation mediates production of PGE2 autacoid to positively regulate interleukin-6 secretion in chronic rhinosinusitis with nasal polyps and polyp-derived fibroblasts
Source: Sci Rep. 2024 Mar 30;14:7559. doi: 10.1038/s41598-024-58143-2 (PMC10981736; doi:10.1038/s41598-024-58143-2)

Supplementary data

Supplementary Table 1 Patients' characteristics.

	Control (n=24)	CRSNP (n=24)	CRSwNP (n=24)	*p* value
**Age, year (min-max)**	37.9±14.3 (17-74)	48.2±12.53 (19-68)	41±16.6 (18-76)	*0.0111 (control *vs* CRSsNP)<br>0.4768 (control *vs* CRSwNP)<br>0.0938 (CRSsNP *vs* CRSwNP)
**Sex (male/female)**	16/8	13/11	15/9	
**Smoker**	16.67%	20.83%	29.17%	
**Reported smell loss**	0%	0%	Total loss (16.67%)<br>Partial loss (41.67%)<br>None loss (41.66%)	
**Asthma**	0%	4.17%	20.83%	
**Atopic status**	0%	4.17%	25.00%	
**CT finding Lund-Mackay score**	Not tested	8.913±4.033 (n=23)	13.42±5.77 (n=24)	**0.0079 (CRSsNP *vs* CRSwNP)
**NP score (sum of left and right nostril scores) (min-max)**	Not tested	Not tested	4.1±1.7 (2-6)	
**SNOT-22 score**	15.92±6.89	27.51±13.61 (n=23)	41.67±13.76	***0.0001 (control *vs* CRSsNP)<br>****<0.0001 (control *vs* CRSwNP)<br>**0.0086 (CRSsNP *vs* CRSwNP)
**Blood eosinophil (%)**	1.367±0.821	2.825±2.673	2.733±2.479	*0.0305 (control *vs* CRSsNP)<br>*0.0136 (control *vs* CRSwNP)<br>0.9878 (CRSsNP *vs* CRSwNP)

*$p<0.05$, **$p<0.01$, ***$p<0.001$, and ****$p<0.0001$ by Mann-Whitney U test.

Materials and Methods

Characterization of prepared human cells

To characterize the NP-derived fibroblasts, cells were seeded on chamber slides and characterized by immunofluorescence microscopy. Briefly, after reaching 80% confluency, cells were washed, fixed with 1% paraformaldehyde (PAF) for 20 min, and permeabilized with 0.1% Triton X-100 for 10 min. Cells were blocked with 3% BSA, incubated with the Ab specific for vimentin (1:200; Santa Cruz Biotechnology), and then followed by the 2nd FITC-conjugated Ab (Chemicon) in the presence of DAPI (1:3000; Thermo Fisher Scientific). After a brief wash, the tissue samples mounted on chamber slides were analyzed under a Nikon Eclipse Ti-S fluorescence microscope (Japan) and photographed using a digital camera.

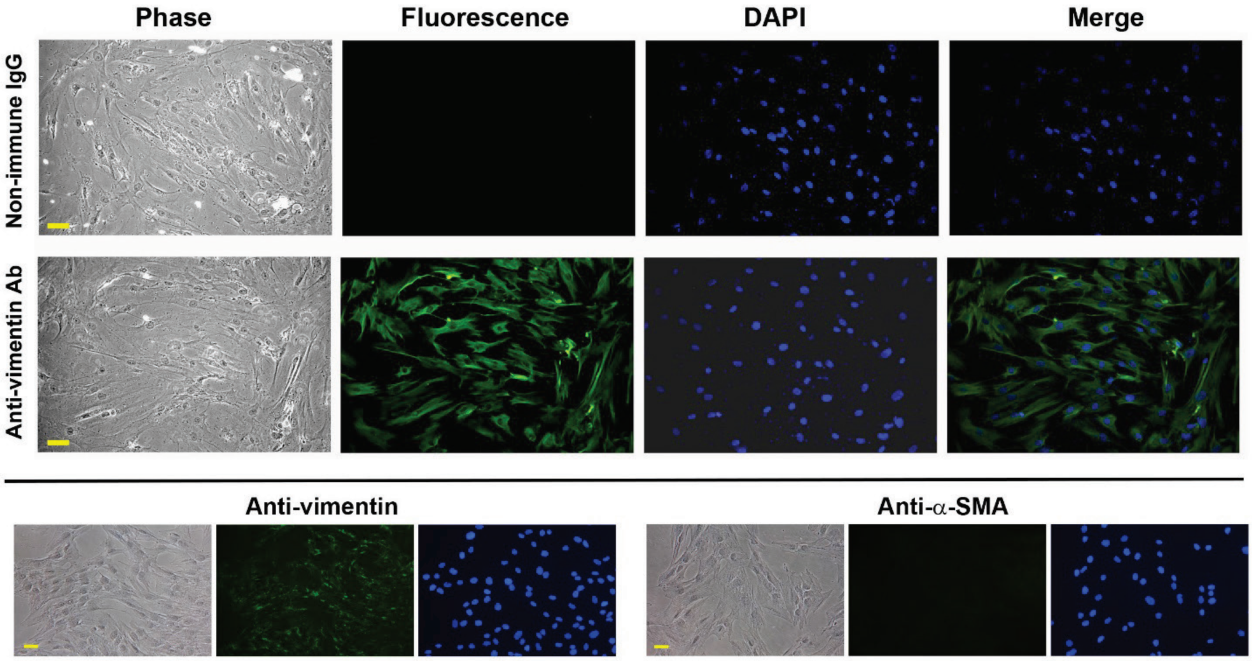

Supplementary Figure 1 Characterization of NP-derived fibroblasts. Cells isolated from CRSwNP NPs were analyzed by ICC. The fixed and permeabilized cells were incubated with the nonimmunized IgG (control) or vimentin Ab (upper panels) or vimentin or α-SMA Ab (lower panels) and followed by the FITC-conjugated secondary Ab and DAPI. Phase: images captured under optical microscopy; Fluorescence: green fluorescence; DAPI: blue fluorescence (nucleus staining); Merge: overlay of two mages with blue and green fluorescence (n=3).

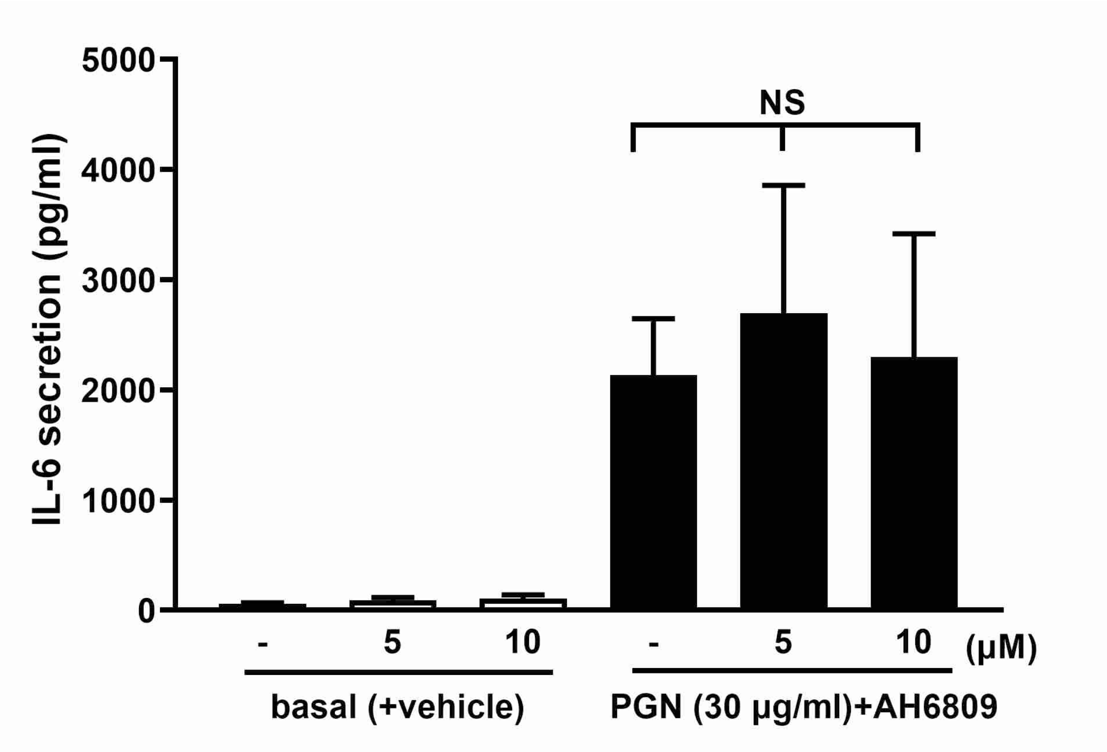

Supplementary Figure 2 Effect of EP1 and EP2 receptor antagonist on PGN-induced IL-6 secretion. The cultured human nasal fibroblasts were treated with vehicle (basal) or PGN in the presence of vehicle (-) or AH6809 (5 or 10 μM) for 16 h. Cell cultured media were collected and IL-6 secretion was analyzed by ELISA. Data are duplicates and are mean±SEM (n=3).

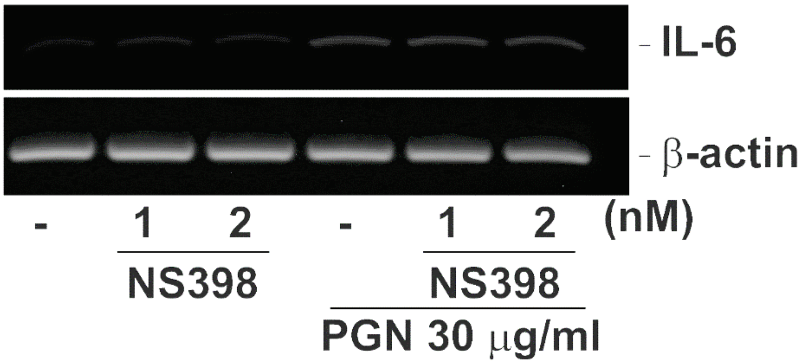

Supplementary Figure 3 Effect of COX-2 inhibitor on PGN-induced IL-6 mRNA expression. The cultured human nasal fibroblasts were stimulated with PGN in the presence of vehicle (-) or NS398 (1 or 2 nM) for 6 h. Cells were collected and prepared and the COX-2 mRNA expression was analyzed immediately by RT-PCR.

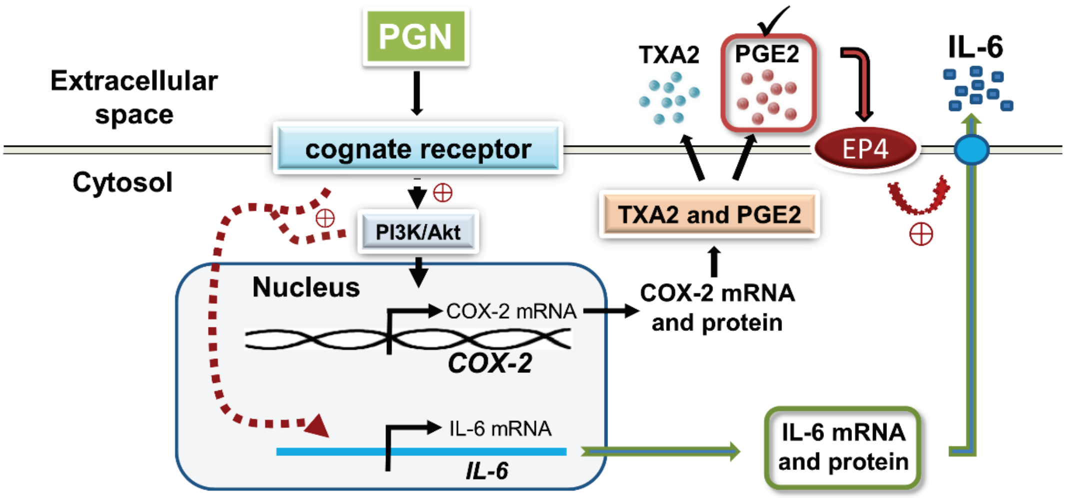

Supplementary Figure 4 The proposed mechanism of the cyclooxygenase-2-mediated interleukin-6 (IL-6) production and secretion in human nasal polyps-derived nasal fibroblasts. The representative peptidoglycan (PGN) binds its cognate receptor and induces PI3K/Akt and cellular signaling and drives *COX-2* and *IL-6* gene transcription, leading to cellular COX-2 mRNA and protein upregulation and subsequently causing PGE_2 and thromboxane A_2 (TXA_2) production and secretion into extracellular space. The PGE_2, but not TXA_2, is likely to activate PGE_2 EP4 receptor in an autacoid way, possibly regulating IL-6 secretion/production via affecting IL-6 protein trafficking. PGN is also likely to cause IL-6 mRNA transcription and protein production/secretion in human nasal polyps-derived nasal fibroblasts (dash line). The Dash lines are putative pathways speculated in this study.

Fig 1_original gel images

Figure 1a

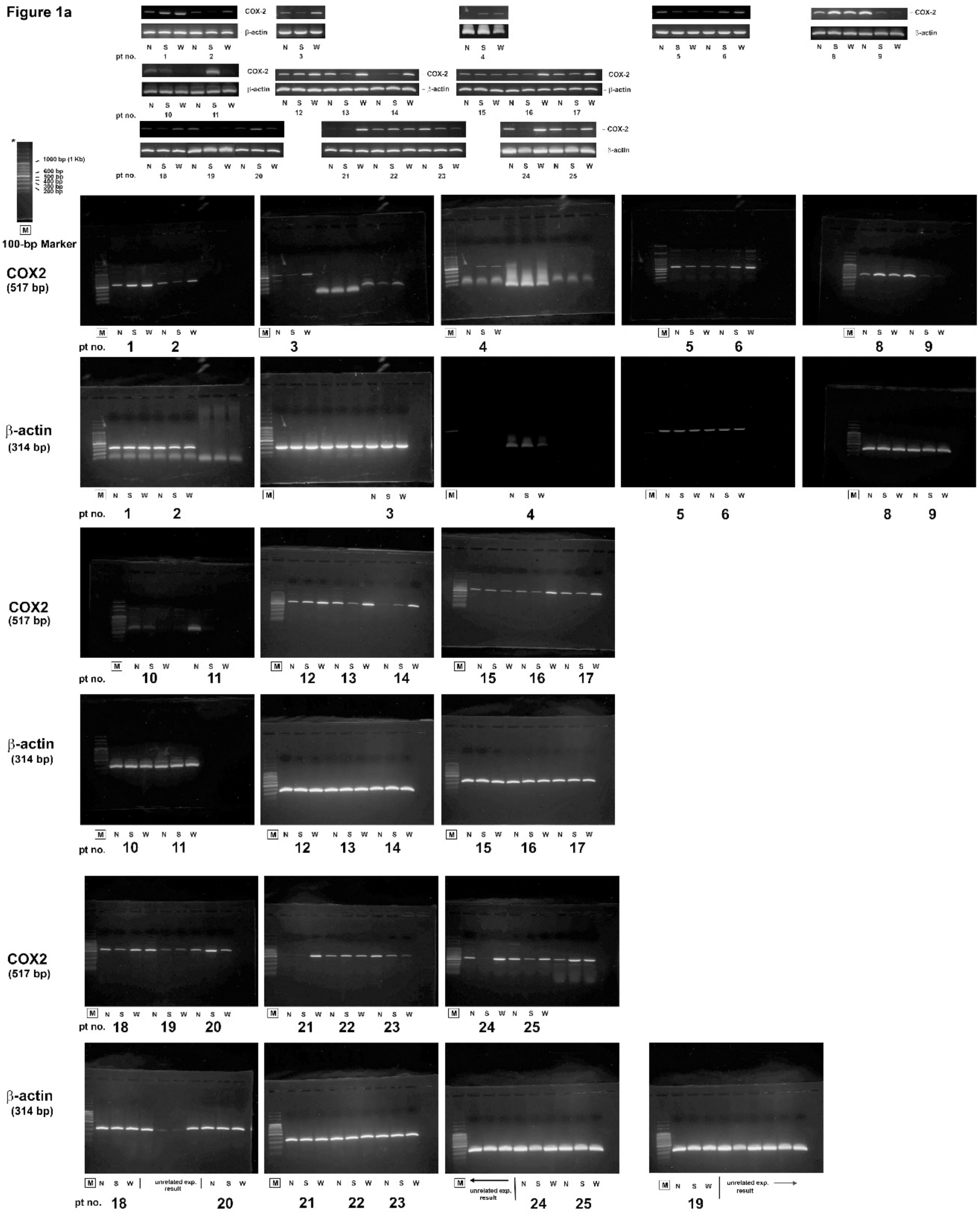

Figure 1b

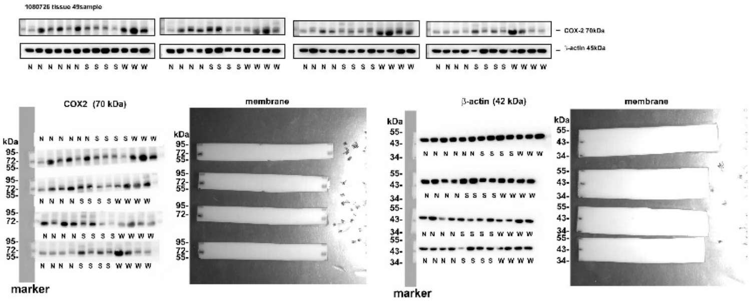

Fig 2a and b_original gel images

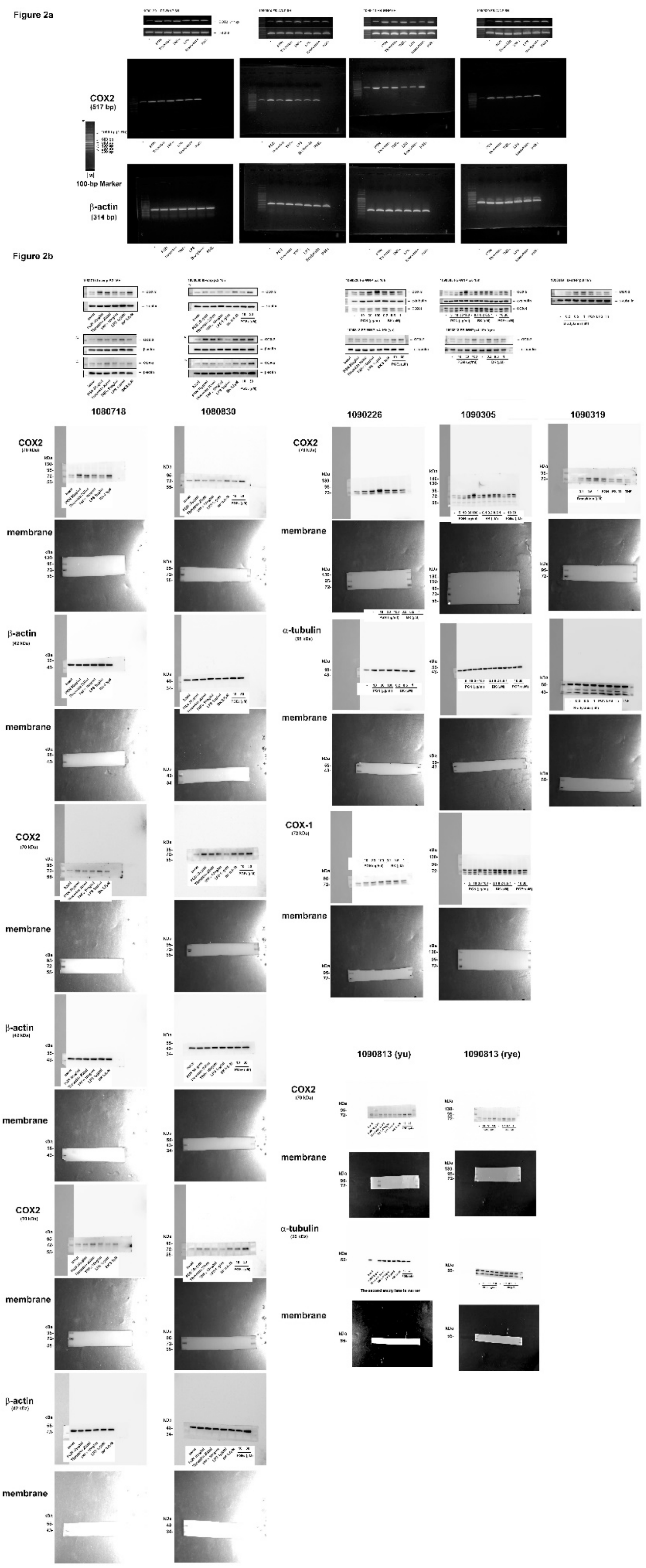

Fig 2c_original gel images

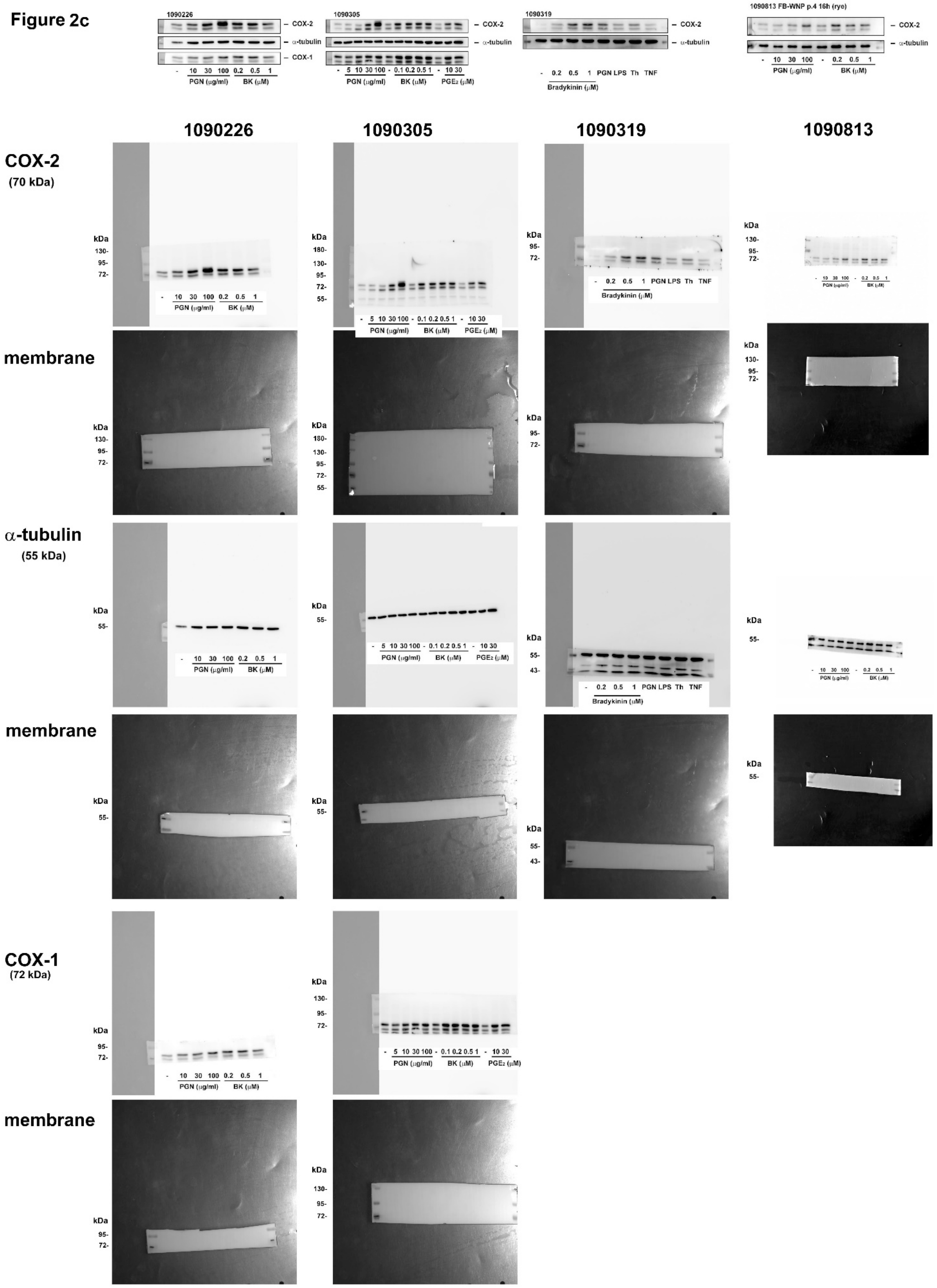

Fig 5b and c_original gel images

Figure 5b

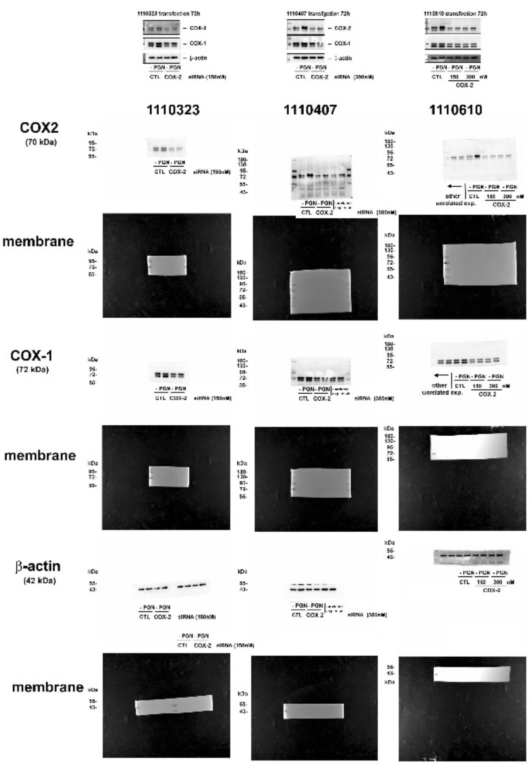

Figure 5c

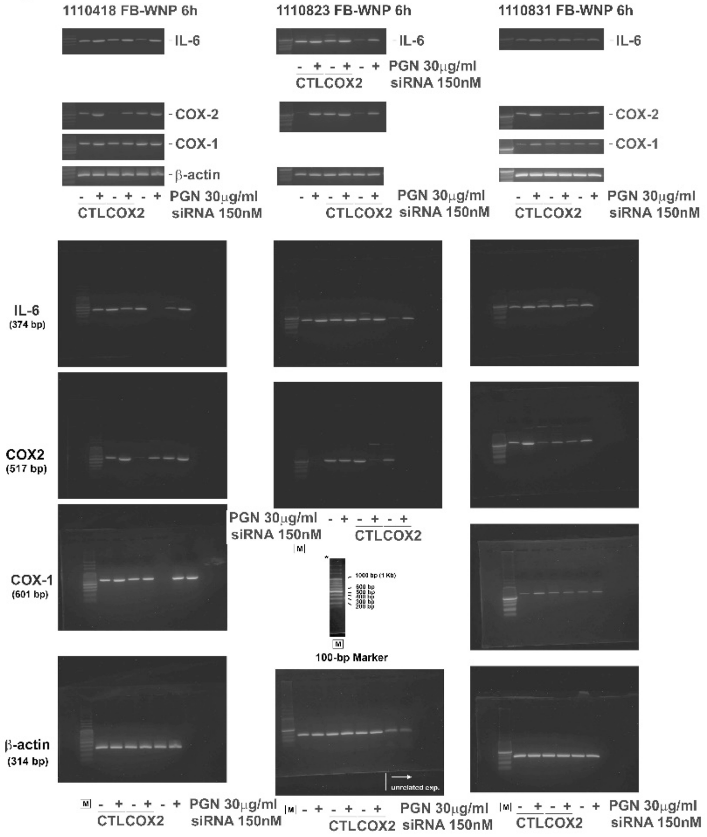

Fig 6a_original gel images

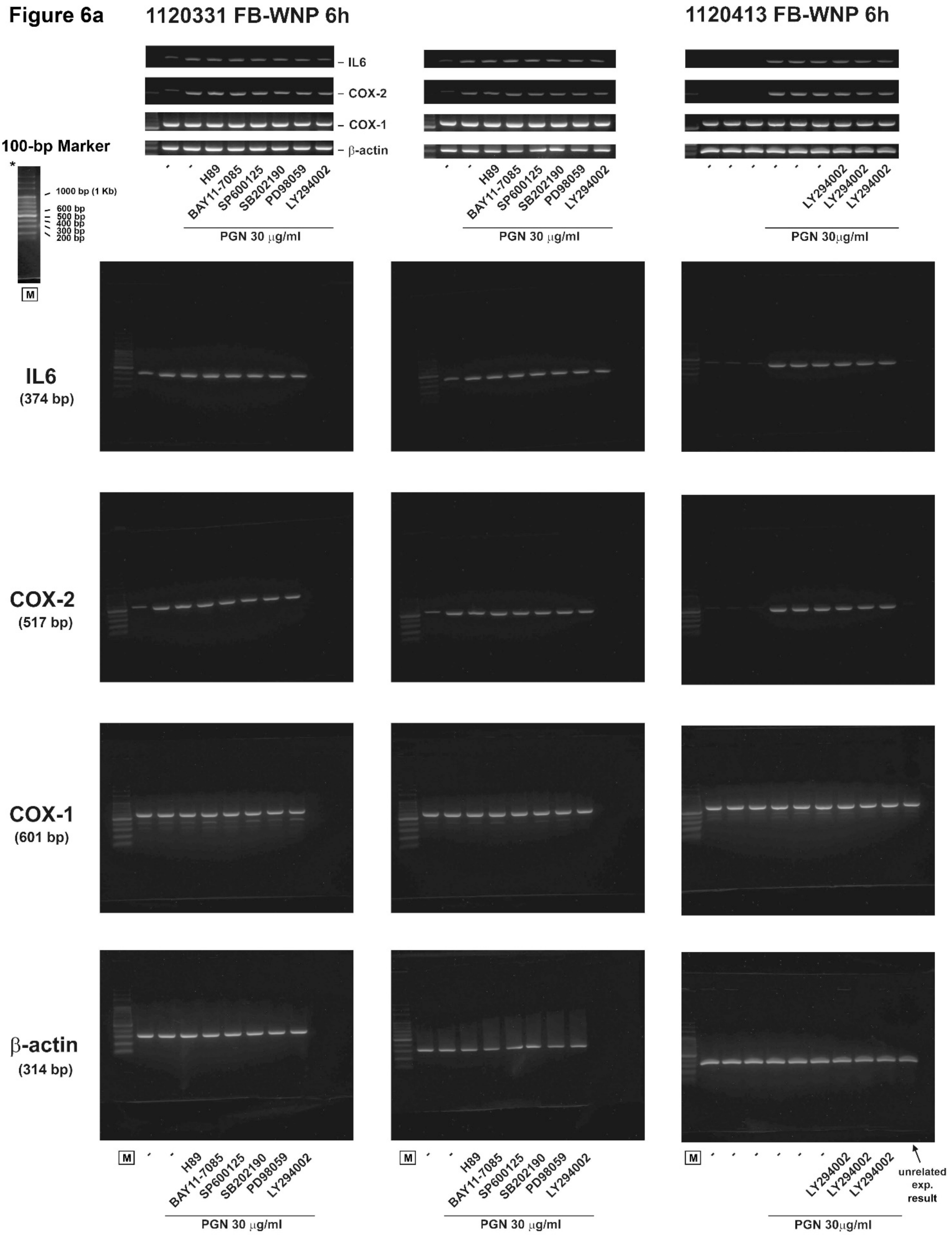

Fig 7_original gel images

Figure 7a

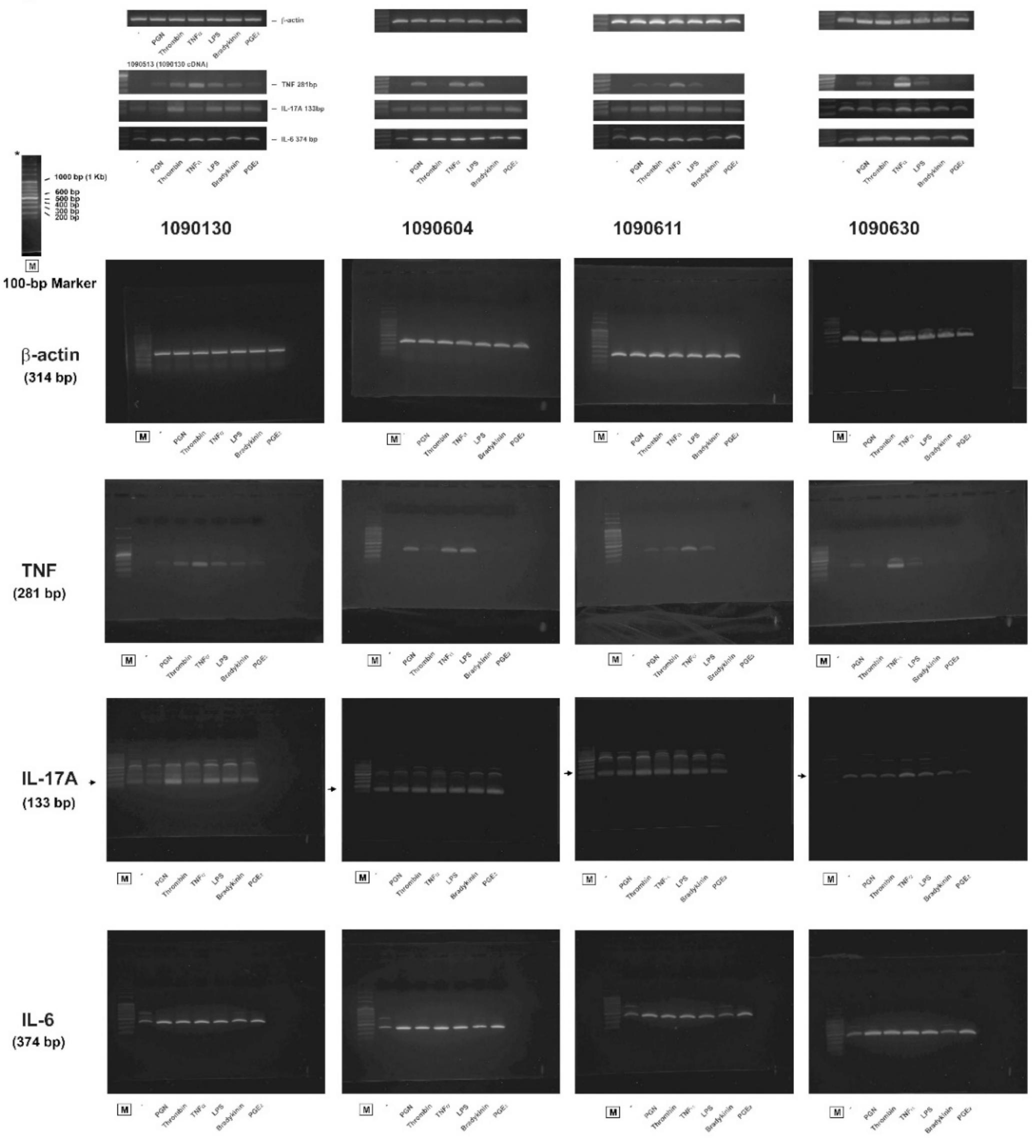

Figure 9b

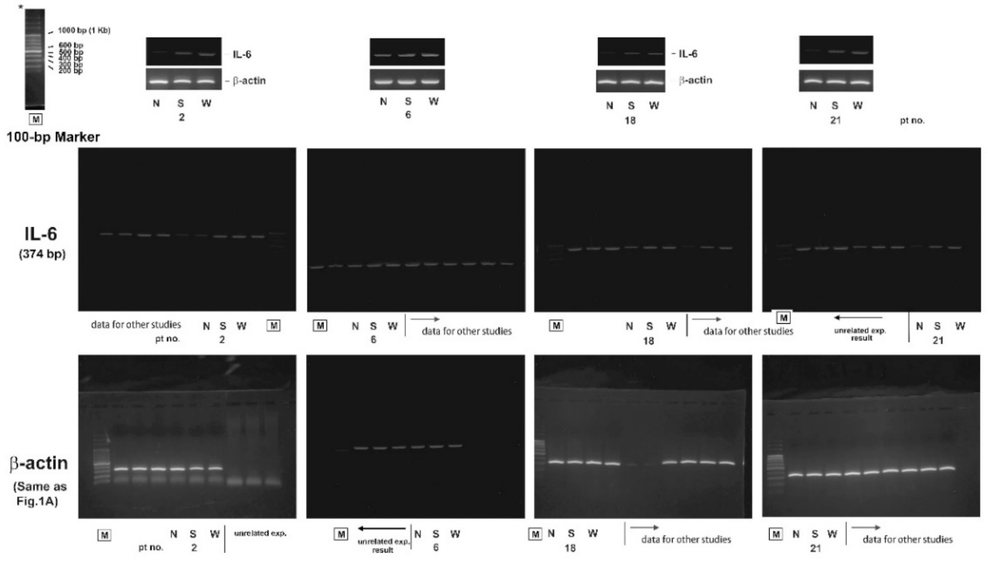

Supp Fig 3_original gel images

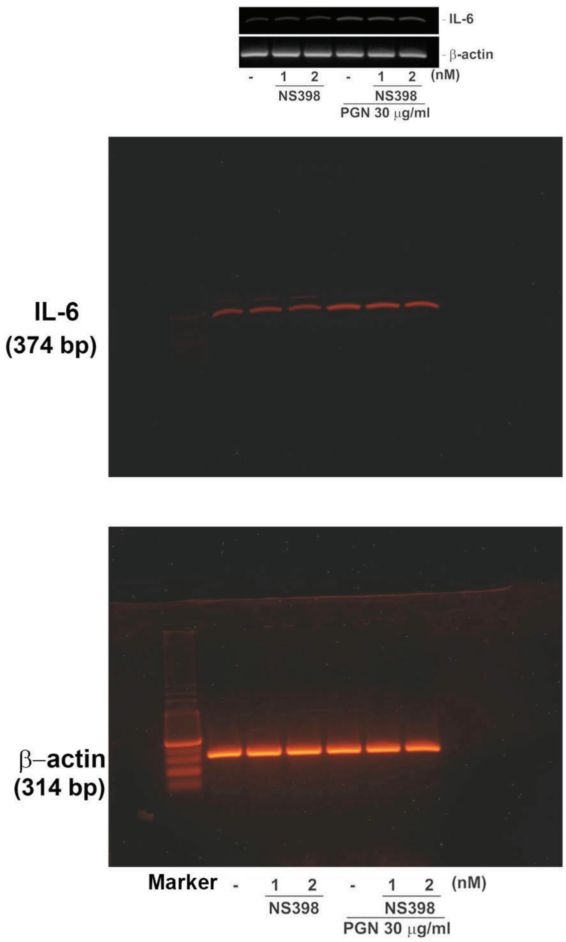

Supplement: Supplementary file 1 — Supplementary Information. [file 41598_2024_58143_MOESM1_ESM.pdf]
